# Synthesis and Characterization of Copper Nanoparticles: A Laboratory Experiment for Undergraduate Physical Chemistry

**DOI:** 10.1021/acs.jchemed.5c00561

**Published:** 2025-11-13

**Authors:** Jonathan Batey, Deborah Okyere, Sarah York, Feng Wang, Jingyi Chen

**Affiliations:** Department of Chemistry and Biochemistry, 3341 University of Arkansas, Fayetteville, Arkansas 72701, United States

**Keywords:** Upper-Division Undergraduate, Physical Chemistry, Interdisciplinary, Problem Solving/Decision Making, Colloids, Metal

## Abstract

Nonprecious metal nanoparticles have been the subject of intensive research, due to their potential applications in promoting sustainability. This integrated laboratory experiment is designed to provide undergraduate students with hands-on experience in synthesizing and characterizing nonprecious metal nanoparticles, specifically copper nanoparticles. The experiment consists of three sections, each tailored to teach a unique aspect of nanochemistry. Section 1 focuses on the air-free synthesis of copper nanoparticles using a solution-based method with an emphasis on understanding their plasmonic properties and how oxidation impacts them. Section 2 explores the quantitative analysis of copper, where students apply crystal field theory to understand the formation of copper-ammonia complexes and their corresponding colors. Section 3 features group presentations and discussions in which students share their results and explore the underlying chemistry through additional materials characterization techniques and density functional theory calculations. By integrating these three sections, this experiment provides a comprehensive teaching tool for undergraduate physical chemistry laboratory courses, promoting student understanding of nanoparticle synthesis, characterization, and applications.

## Graphical abstract

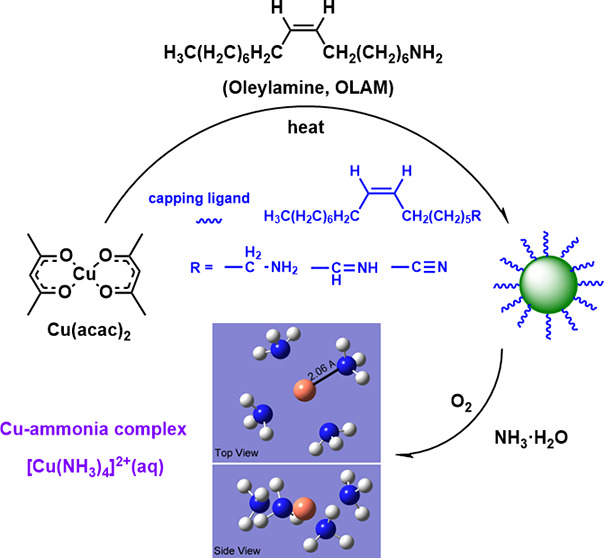

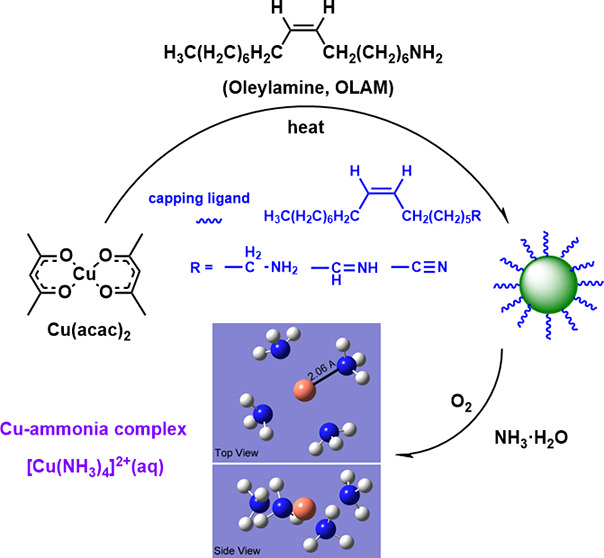

## Introduction

Earth-abundant metal nanoparticles have drawn considerable attention in various applications related to chemical and energy conversion, because their abundance and low cost make them suitable for developing commercially viable advanced technologies. Among them, Cu nanostructures are of particular interest due to their high electrical conductivity, strong optical properties in visible and near-infrared regions, and excellent catalytic properties, rendering them suitable for a wide variety of applications in flexible electronics, photonics, and catalysis.^1–3^ Based on recent research advancements, several educational experiments have been developed for use in laboratory teaching. For example, Cu was used in an experiment where it was mixed with wax to simulate the electrical behavior of metal-filled plastics.^4^ The synthesis of dendrimer-encapsulated Cu nanoparticles and their catalytic evaluation using *p*-nitrophenol reduction were integrated into a laboratory experiment.^5^ The optical properties of Cu nanoparticles were presented in a series of redox reactions to demonstrate the color changes and reactivity at the nanoscale.^6^ A chemically etched, nanostructured Cu was used for surface-enhanced Raman spectroscopy.^7^ However, there are still few experiments demonstrating the impact of air-free chemistry on Cu nanoparticle synthesis, optical properties, and reactivity to ligands.

We present an integrated experiment for undergraduate laboratory teaching that focuses on the synthesis and characterization of Cu nanoparticles. Simplified airtight methods are employed for the synthesis. The formation of these nanoparticles is evaluated by their optical properties, specifically localized surface plasmon resonance (LSPR), and the Cu concentration is quantified via colorimetry based on the formation of Cu-ammonia complexes. An oleylamine (OLAM)-mediated method is employed for the synthesis of Cu nanoparticles. Since OLAM is commonly used in oil-based syntheses of inorganic nanoparticles,^8,9^ the experiment, using Cu as an example, can help students understand the role of OLAM in the synthesis of metal nanoparticles. Our group has been employing the OLAM-mediated method to synthesize Cu nanostructures and their alloys with Au, Pd, Pt, and Ru.^10–13^ In this experiment, students synthesize Cu nanoparticles and use UV–vis spectroscopy to determine their success based on optical properties. They also learn complementary techniques, transmission electron microscopy (TEM), and X-ray diffraction (XRD), by using free resources to analyze TEM images and XRD patterns to determine particle size, index XRD patterns, and validate the UV–vis measurements. Furthermore, they quantify the Cu concentration in nanoparticle suspensions using colorimetry based on crystal field theory by dissolving nanoparticles with ammonia to form a Cu-ammonia complex. Density functional theory (DFT) calculations help students understand the origin of the complex’s color. This experiment provides a comprehensive introduction to nanochemistry from physical and materials chemistry perspectives.

## Experimental Overview

Upper-level chemistry students learn an air-free synthesis of Cu nanoparticles and their optical properties, as well as a colorimetric method for Cu quantification and crystal field theory through a three-section laboratory experiment (Table 1). Full details of the experiment can be found in the .

**Table 1:** Summary of Activities in the Laboratory

section	content	time required	Supporting Information
1	Synthesis and Optical Characterization of Cu Nanoparticles	4 h	
			
2	Quantification of Cu Concentration via a Colorimetric Method	4 h	
			
3	Data Analysis and Group Discussion	3 h	
	Demo of DFT Calculation	1 h	

Section 1 focuses on the synthesis of Cu nanoparticles and their optical characterization, with an emphasis on teaching students the crucial role of air-free chemistry in synthesizing nonprecious metal Cu, as well as the impact of oxidation on the plasmonic properties of these nanoparticles. The reaction scheme is illustrated in Figure 1. The reduction of Cu­(acac)_2_ by OLAM can be presented by the following chemical equation (eq 1).

$$\mathrm{Cu}{\left(O_{2}C_{5}H_{7}\right)}_{2}\left(s\right)+C_{18}H_{35}{\mathrm{NH}}_{2}\left(l\right)\to \mathrm{Cu}\left(s\right)+2C_{5}H_{7}O_{2}H\left(l\right)+C_{17}H_{33}\mathrm{CH}\mathrm{=NH}\left(l\right)$$ 1

The role of OLAM is both that of a reducing agent and a stabilizer in the reaction.^8^ The reaction involves the reduction of Cu­(II) to Cu­(I) and then Cu(0) through the formation of Cu­(II)-OLAM and Cu­(I)-OLAM.^14^ The electron transfer from OLAM to Cu­(II) and Cu­(I) to form Cu(0) at an elevated temperature may involve amino radicals as intermediates, similar to the reduction of Ag­(I) to Ag(0) by OLAM.^15^ These amino radicals could be further oxidized to imine and nitrile, becoming part of the capping ligands for the Cu nanoparticles, which could be analyzed by attenuated total reflectance Fourier transform infrared spectroscopy (ATR-FTIR).^16^ After completion of the reaction, students evaluate the success of their synthesis by measuring the LSPR properties of the nanoparticles, using UV–vis spectroscopy.

**Figure 1:**
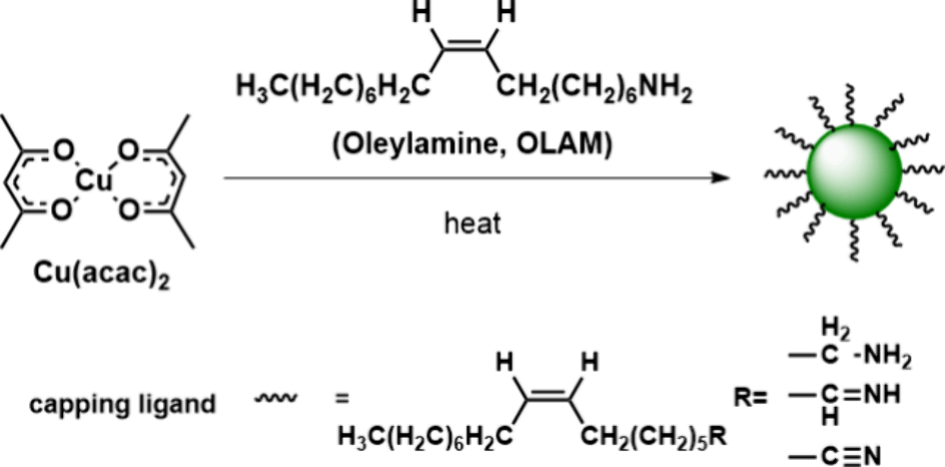
Schematic illustration of the formation of Cu nanoparticles by the OLAM-mediated reduction of the Cu precursor under air-free conditions.

Section 2 emphasizes quantification of Cu concentration using a colorimetric method based on Cu-ammonia complexes, with the goal of teaching students about the reactivity of Cu with ligands and introducing them to the fundamental principles of crystal field theory. The mechanism of Cu dissolution in ammonia solutions was suggested by Halpern in 1953 ^17^ and further investigated by others for chemical mechanical polishing.^18^ The dissolution process involves oxygen annd is described by eq 2:

$$\mathrm{Cu}\left(s\right)+\frac{1}{2}O_{2}\left(g\right)+4{\mathrm{NH}}_{3}\cdot H_{2}O\left(l\right)\to {\left[\mathrm{Cu}{\left({\mathrm{NH}}_{3}\right)}_{4}\right]}^{2+}\left(\mathrm{aq}\right)+2{\mathrm{OH}}^{-}\left(\mathrm{aq}\right)+2H_{2}O\left(l\right)$$ 2

The dissolution kinetics for the bulk of Cu are dependent on the transport of oxygen, whereas this is significantly less of a concern for Cu nanoparticles, due to their high surface-to-volume ratio. Students dissolve Cu nanoparticles in ammonia solutions, establish calibration curves, determine Cu concentrations, and calculate the reaction yields of the synthesis in Section 2.

Section 3 features group presentations and discussions, designed to facilitate student interaction and the sharing of experimental findings, while also introducing them to materials analysis and theoretical simulations. Group presentations (12 min + 3 min Q&A) are followed by a class discussion on how TEM, XRD, and UV–vis spectroscopy complement each other in characterizing Cu nanoparticles. Students also engage in hands-on practice with ImageJ software^19^ for TEM image analysis and XRD pattern indexing^20,21^ (sample data included in the ), and participate in a DFT calculation demonstration to understand the origin of the Cu-ammonia complex’s color.

## Chemicals

All chemicals were used as received (see ). If H_2_O was used in the experiments, it refers to deionized H_2_O with a resistivity of 18 MΩ. Stock solutions were prepared by the teaching assistant prior to the class (see the ).

## Hazards

Chemicals such as toluene, ethanol, and ammonia–water can cause skin irritation, eye damage, and respiratory problems and are toxic and highly flammable. When the experimental procedures are conducted, lab coats, gloves, and safety goggles should be worn. Volatile substances should be handled in well-ventilated areas or in fume hoods. Precaution should be exercised during high-temperature operations.

## Results and Discussion

### Section 1. Synthesis of Cu Nanoparticles

Students carried out the synthesis of Cu nanoparticles under inert gas protection in a fume hood using simplified airtight setups with a single manifold. A representative setup is shown in Figure 2A, where a thermocouple is inserted into the reaction solution to directly monitor and control the reaction temperature throughout the experiment. During the reduction of Cu­(acac)_2_ by OLAM, students observed a series of the color changes (Figures 2B–E), which progressed from green, to dark green, yellowish dark, and brownish dark, indicating the conversion of Cu­(II) to Cu(0).

**Figure 2:**
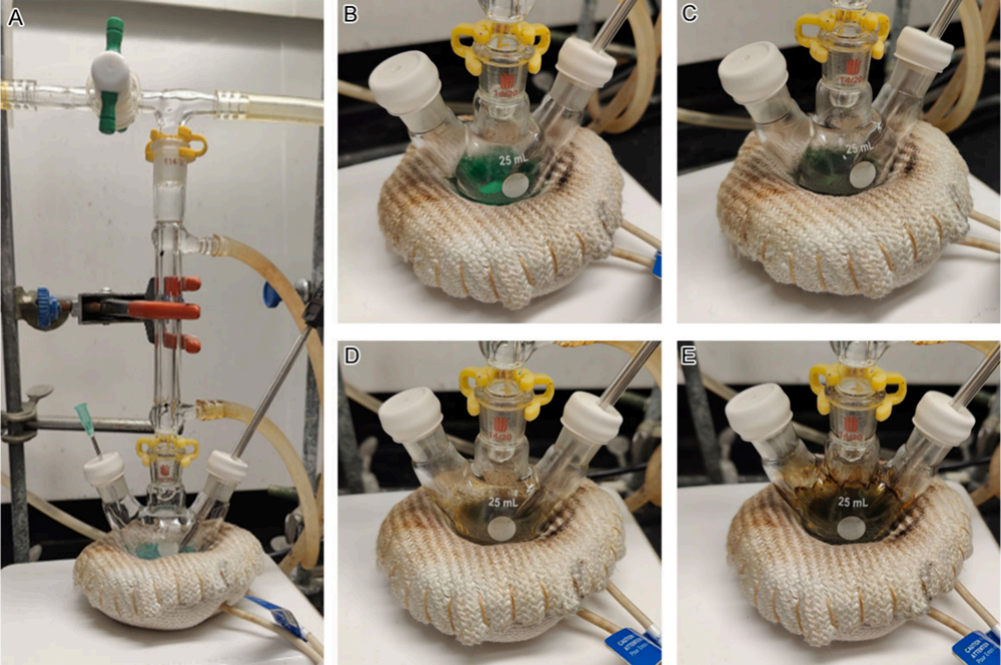
Photographs of (A) the reaction setup and (B–E) the color changes of the reaction solution. The precursor was degassed in the reaction flask and appeared as a blue solid. After degassing, the needle was removed, and the valve was turned to the horizontal position to maintain an inert atmosphere for the reaction. As the reaction solution was heated from 150 °C to 220 °C over 10 min, its color changed sequentially from green (panel (B)), to dark green (panel (C)), yellowish dark (panel (D)), and finally to brownish dark (panel (E)).

Following completion of the reaction, the mixture was allowed to cool to room temperature (Figure 3A). The reaction mixture was then transferred to a 50 mL centrifuge tube, and the Cu nanoparticles were collected by centrifugation. They were purified by repeated washing with a toluene/ethanol mixture twice. Figure 3B shows images of the mixture before and after centrifugation, highlighting separation of the supernatant. Prior to centrifugation, the mixture appeared dark. Following separation, the supernatant was yellowish brown, while the precipitate was dark blue. The purified nanoparticles were redispersed in toluene, as in Sample 1 (Figure 3C). The characteristic dark blue color of the particle suspension became more apparent upon dilution, corresponding to the LSPR peak of the Sample 1 Cu nanoparticles at 590 nm in their UV-vis spectrum (Figure 3D).

**Figure 3:**
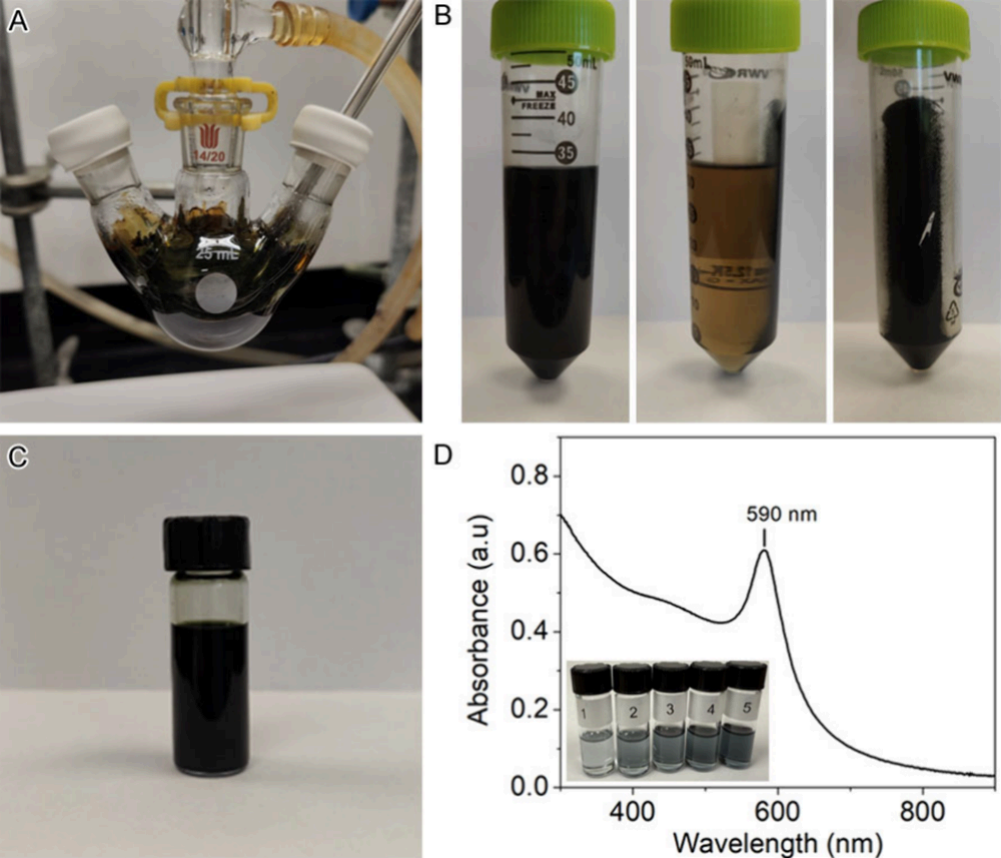
(A) Photograph documenting the reaction mixture cooling to room temperature. (B) Images of the mixture before and after centrifugation, with the supernatant removed. (C) Photograph of the final, purified Cu nanoparticles (Sample 1) resuspended in toluene. (D) UV-vis spectrum of a diluted Sample 1 nanoparticle suspension in toluene, with the inset showing a series of dilutions.

The LSPR peak is a collective oscillation of electrons at the surface of metal nanoparticles, which is sensitive to the particle size, shape, and composition.^2^ In this case, the sharp and strong LSPR peak indicates that the students successfully synthesized metallic Cu nanoparticles with limited surface oxidation. Oxidation can lead to damping of the LSPR peak due to the formation of an oxide layer on the surface of the nanoparticles, resulting in a broadened or weakened LSPR peak. The presence of a sharp and strong LSPR peak at 590 nm suggests that the Cu nanoparticles have a relatively clean surface with minimal oxidation, allowing for a strong collective oscillation of electrons and an intense absorption and scattering of light at this wavelength.

Students further verified the formation of Cu nanoparticles in Sample 1 by TEM and XRD, for which the data was collected by their teaching assistant. The TEM image in Figure 4A shows a 2D projection of the sample from which it can be inferred that the nanoparticles are roughly spherical in morphology. Students analyzed the particle size using ImageJ software in Section 3. Since aggregates observed in TEM images may be an artifact of sample preparation, we advise students to select only well-resolved, nonoverlapping particles for size analysis. Based on the ImageJ analysis of Figure 4A, the nanoparticles have an average diameter of 15.4 ± 3.4 nm. The XRD pattern of the nanoparticles in Figure 4B can be indexed to the face-centered cubic (FCC) crystal structure of Cu (amcsd#0011145).^20,21^ The observed peaks at 43.35°, 50,49°, 74.20°, and 89.98° (2θ) can be assigned to the (111), (200), (220), and (311) crystallographic planes of the Cu FCC structure, respectively. The XRD results confirm that the nanoparticles are made of metallic Cu.

**Figure 4:**
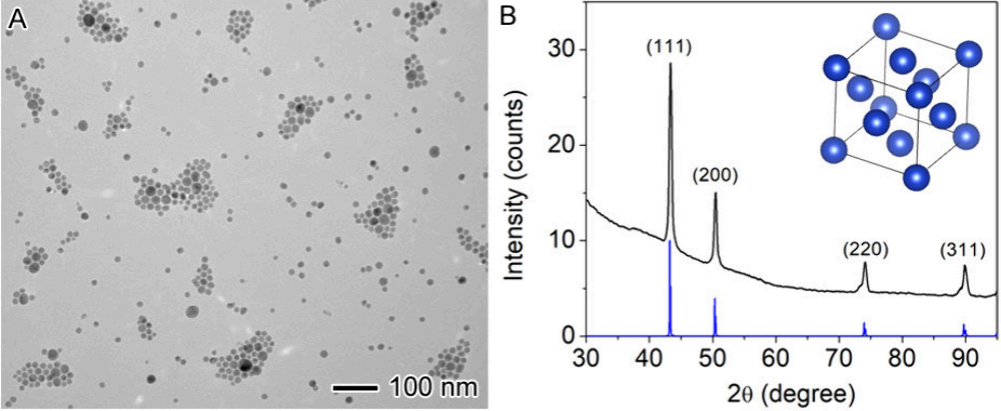
Characterization of the Cu nanoparticles Sample 1 synthesized by reducing Cu­(acac)_2_ in the presence of OLAM: (A) TEM image and (B) XRD with an inset of the fcc unit cell. The observed XRD pattern (black) is indexed to the corresponding to that of Cu fcc structure (blue).

Students also conducted syntheses in an airtight vessel heated by a mantle (Figure 5A), a setup that eliminates the need for a temperature probe immersed in the solution. This configuration makes the experiment readily adaptable to various laboratory environments. However, prior to the reaction, temperature calibration and inert gas purging were necessary steps that were carried out by a teaching assistant. A thermometer was used to calibrate the solution temperature (Figure 5B), revealing a 50 °C discrepancy between the set heat mantle temperature and actual solution temperature. For instance, a set temperature of 270 °C was required to achieve a reaction temperature of 220 °C measured in the solution. Note that the reaction temperature refers to the solution temperature. Before the reaction was initiated, argon purging removed air from the mixture (Figure 5C). Inadequate heating or residual air can result in the formation of oxidative byproducts.

**Figure 5:**
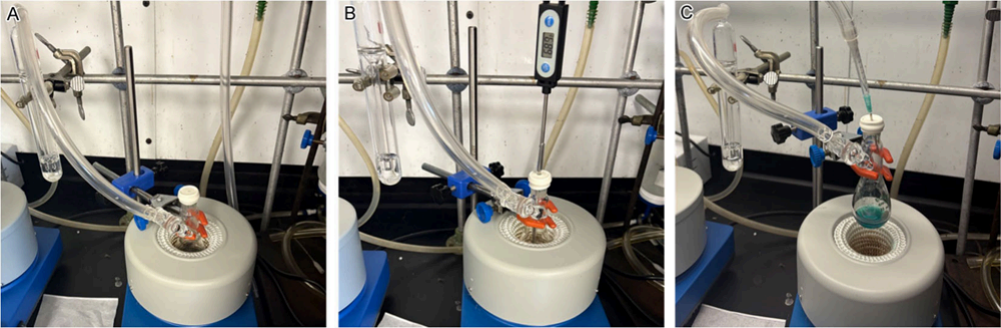
Photographs of the simplified airtight vessel used for the synthesis of Cu nanoparticles: (A) reaction in progress; (B) solution temperature measurement; and (C) argon purging.

Representative results from student groups are presented in Figure 6, illustrating nanoparticle products from reactions with varying Cu_2_O to Cu ratios. Sample 2, synthesized at 180 °C, without argon purging, indicated a complete loss of the plasmonic properties of Cu (Figure 6A), whereas sample 3, obtained at 220 °C, with argon purging, exhibited a broaden and weaken plasmonic peak, compared to that of Sample 1 (Figure 6D). XRD measurements confirmed that the optical properties depend on the compositions of the nanoparticles. TEM characterization revealed distinct morphological differences between the two samples. The former exhibited a characteristic Cu_2_O pattern (Figure 6B) with dendritic growth (Figure 6C), whereas the latter showed a dominant Cu pattern (Figure 6E) with a reduced dendritic morphology (Figure 6F). These results underscore the importance of inert gas protection during synthesis, as oxidation can dampen the plasmonic properties of Cu nanoparticles.

**Figure 6:**
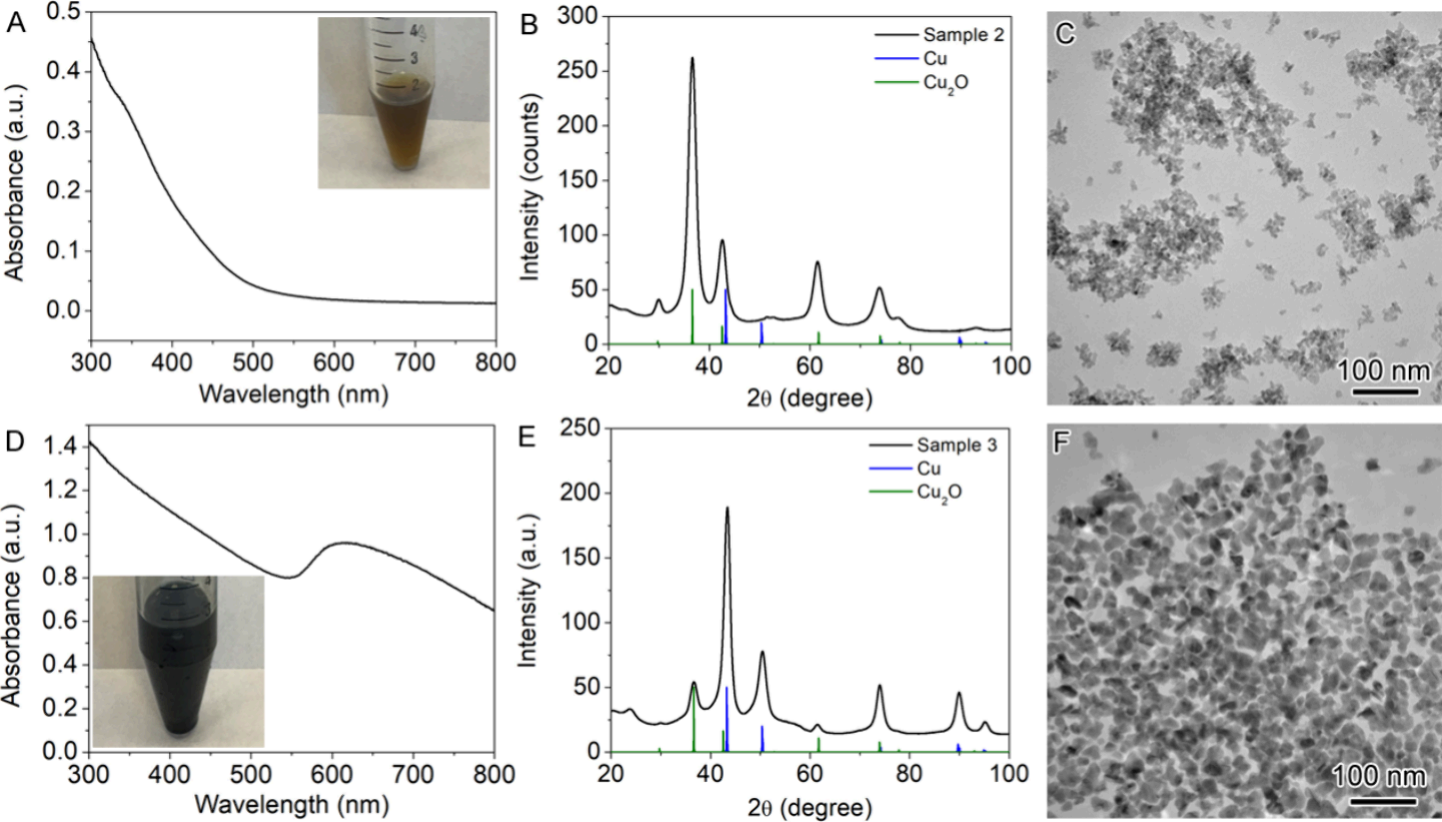
Characterization of Samples 2 and 3 obtained from the simplified airtight setup synthesized at different reaction conditions: (A–C) 180 °C, without argon purging, and (D–F) 220 °C, with argon purging.

### Section 2. Quantification of Cu Concentration via a Colorimetric Method

Students determined the Cu concentration in the nanoparticle suspension using a colorimetric method based on the formation of a Cu-ammonia complex, [Cu­(NH_3_)_4_]^2+^. The procedure involved drying a 200 μL aliquot via a steady nitrogen stream and preparing working solutions from a stock solution (25 mg of CuCl_2_ in 10 mL of NH_3_·H_2_O/H_2_O mixture) to establish a calibration curve. A typical set of working solutions is shown in Figure 7A, with their UV–vis spectra displayed in Figure 7B. The calibration curve can be plotted by using the absorbance at 600 nm of working solutions versus their concentration, as shown in Figure 7C. The linear fit of the calibration curve yields the equation *y* = 42.7*x* (*R* ^2^ = 0.999), where the molar absorptivity of the Cu-ammonia complex can be determined from the slope of the curve, in this example, corresponding to be 42.7 M^–1^ cm^–1^. To ensure accurate results, students waited 15 min before taking optical measurements, as validated by the stability assessment in Figure 7D, which showed no change after 15 min.

**Figure 7:**
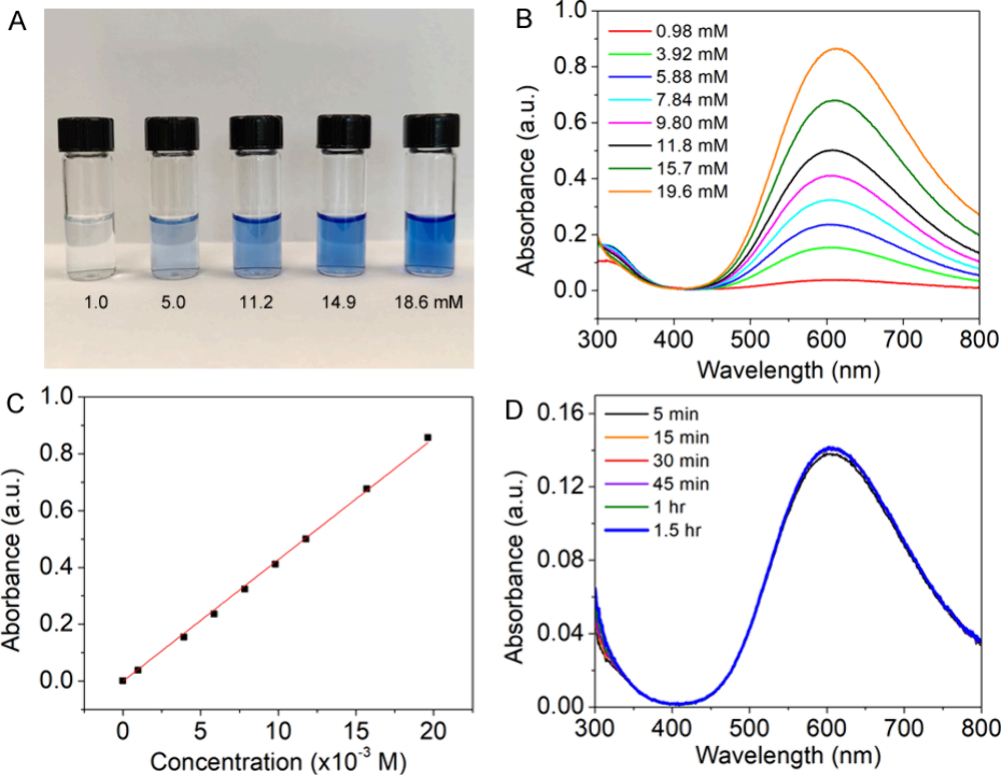
Quantification of Cu concentration using a colorimetric method involving the formation of a Cu-ammonia complex: (A) photograph of a dilution series, (B) UV-vis spectra of the standard solutions, (C) plot of the calibration curve, and (D) UV–vis spectra of the time course study of the Cu nanoparticles dissolution in a NH_3_·H_2_O/H_2_O mixture.

Students dissolved their dried nanoparticles in 200 μL of NH_3_·H_2_O, and then they added water (1.2–1.8 mL) to achieve the desired concentration. After 15 min, they measured the UV–vis spectra of the solutions (Figure 8). The intensity at 600 nm was used to compute the Cu concentration using the molar absorptivity determined from their calibration curves. For examples, the Cu concentrations of Samples 1–3, were calculated to be 18.4, 9.0, and 15.8 mM, respectively, based on the molar absorptivity of 42.7 M^–1^cm^–1^ and the dilution factor for each sample.

**Figure 8:**
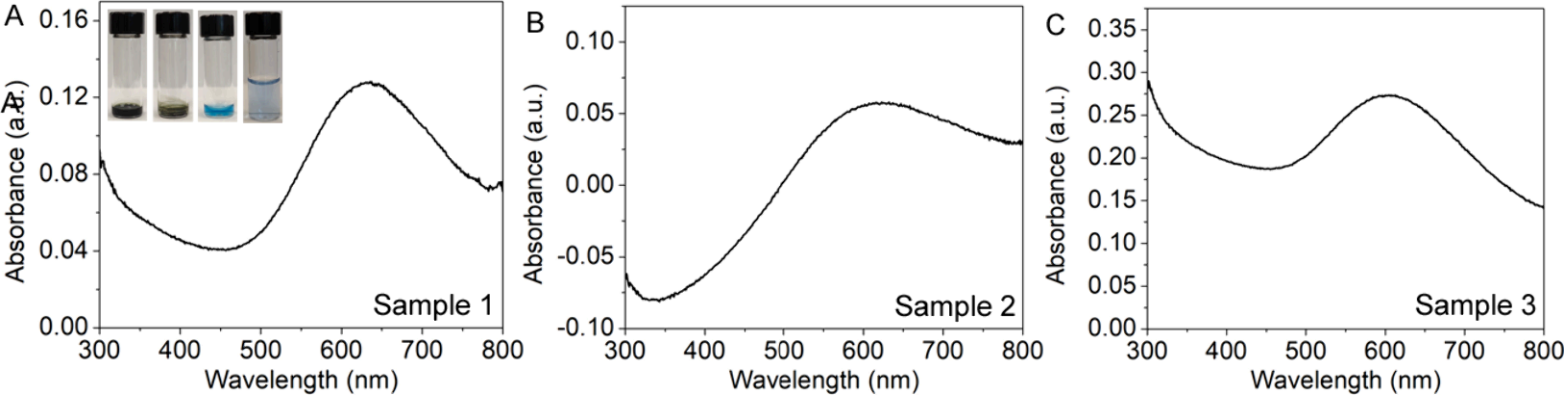
UV–vis spectra of the dried samples dissolved in NH_3_·H_2_O and diluted to the desired volume: (A) Sample 1, (B) Sample 2, and (C) Sample 3. Photographs from left to right showing 200 μL aliquot of nanoparticle suspension, dried nanoparticles, dissolved nanoparticles in 200 μL of NH_3_·H_2_O, and its subsequent dilution before the UV-vis measurement was taken.

Additionally, students could calculate the reaction yield of Cu nanoparticle synthesis. If the volume of the particle suspension is known, then one can determine the yield of the reaction using the total Cu amount of the Cu nanoparticle suspension. For example, students accurately dispersed the Cu nanoparticles in 3 mL of toluene after the purification. The amounts of Cu in Samples 1–3 were calculated as 0.056, 0.027, and 0.047 mmol, based on their concentrations (18.4, 9.0, and 15.8 mM) in 3 mL suspensions. Compared to the 0.2 mmol Cu­(acac)_2_ added, the yields were 28.0%, 13.5%, and 23.5% for Sample 1–3, respectively. Notably, Sample 2’s lower yield is attributed to its lower reaction temperature.

### Section 3. Data Analysis and DFT Demonstration

The first part primarily focuses on estimating the concentration of metal nanoparticles using results from Sections 1 and 2. The calculation method, applicable to metallic nanoparticles with FCC structures, provides a general overview of the particle concentration estimation. The particle concentration (*C*_particle_, particles/mL) can be calculated using eq 3:

$$C_{\mathrm{particle}}=C_{\mathrm{Cu}}/M_{\mathrm{CuNP}}$$ 3

where *C*_Cu_ is the concentration of elemental Cu and *M*_CuNP_ is the mass per Cu nanoparticle. *M*_CuNP_ can be calculated based on TEM image analysis, as detailed in . Equations 4–6 provide additional steps for calculating the particle volume, mass, and molar concentration. The volume of a spherical particle (*V*, nm) with a radius of *r* (nm) can be obtained by eq 4:

$$V=\frac{4}{3}\pi r^{3}$$ 4

The mass per Cu nanoparticle (*M*_CuNP_, g) can then be calculated using eq 5:

$$M_{\mathrm{CuNP}}=\frac{V}{a^{3}}\times N\times M_{\mathrm{Cu}}$$ 5

where *a* is the lattice constant of Cu (0.361 nm),^20,21^ *N* is the number of atoms per FCC unit cell (4 atoms), and *M*_Cu_ is the atomic mass of Cu (63.5 amu or 1.055 × 10^–22^ g per atom) . The particle concentration can be converted to molar concentration (*C*_M,particle_) using eq 6:

$$C_{M,\mathrm{particle}}=C_{\mathrm{particle}}/N_{a}$$ 6

The second part comprises a demonstration of DFT calculations to elucidate the structural and optical properties of [Cu­(NH_3_)_4_]^2+^. The moderate crystal field splitting energy due to the ammonia ligands leads to a square planar geometry, as opposed to a tetrahedral or octahedral arrangement. This geometry is favored, because it minimizes electron pairing and maximizes orbital overlap between Cu^2+^ and NH_3_. The resulting d-orbital splitting gives rise to characteristic d–d transitions in the visible region, which are responsible for the complex’s blue color and can be used to probe its electronic structure and bonding properties. Figure 9A shows a square planar configuration of [Cu­(NH_3_)_4_]^2+^ with two loosely bound water forming a highly distorted octahedron optimized using DFT. The loosely bound water is not included in the model. The DFT calculation is performed using Gaussian 16 with the B3LYP exchange correlation functional^22^ and the aug-cc-pVDZ basis set.^23,24^ The Cu–N distances from B3LYP optimization is 2.06 Å, which is very close to the experimentally found value of 2.05 Å.^25^

**Figure 9:**
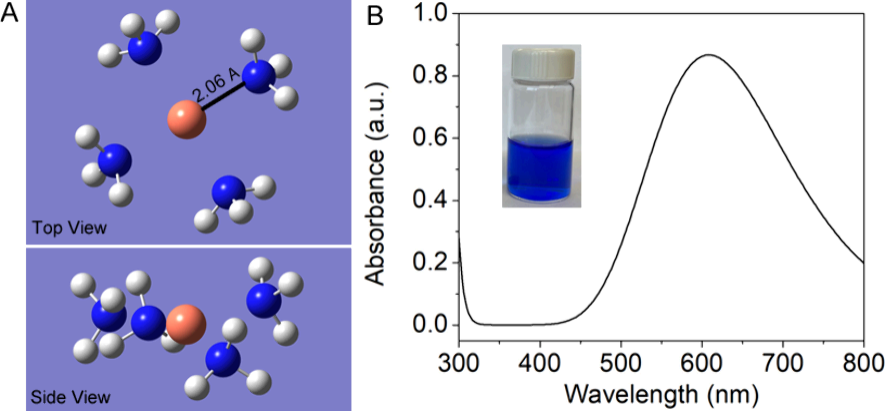
(A) Top and side view of the [Cu­(NH_3_)_4_]^2+^ conformation, optimized using DFT, revealing the buckling of a square planar geometry that breaks the symmetry. (B) Calculated optical spectrum of the [Cu­(NH_3_)_4_]^2+^ complex. The inset displays an aqueous solution of the complex.

A time-dependent (TD)-DFT calculation was also performed with Gaussian 16. The TD-DFT shows a very weak transition at 605 nm (Figure 9B), consistent with the measurements. The d–d transition is Laporte-forbidden for molecules with a center of symmetry, but it is weakly allowed for the Cu-ammonia complex due to strong Jahn–Teller distortion of the octahedron formed by the NH_3_ and H_2_O molecules.^26^ For the DFT-optimized structure, the square formed by the four NH_3_ atoms is slightly buckled, due to repulsion between the H atoms, breaking the center of symmetry. This explains the substantially larger extinction coefficient of Cu-ammonia complex when compared to the Cu-aquo complex, which has a much weaker Jahn–Teller distortion. It is worth noting that the 600-nm adsorption is in the red region of the visible spectrum. The blue of the solution is its complementary color. This phenomenon is also illustrated in an educational video titled “Copper” published on the YouTube channel “Chemistry_Rules” by the graduate students of physical chemistry at the University of Arkansas.^27^

### Formative Assessment

Formative assessment played a crucial role in evaluating student learning throughout the laboratory experiment, using multiple methods that combined short quizzes, group presentations and discussions, and laboratory reports. As the study fell under a general institutional protocol for classroom activities, a formal ethics review was not possible.

To ensure students’ preparation for lab experiments, brief 15 min quizzes at the beginning of Sections 1 and 2 were administered. Prelab quizzes (samples included in ) are designed to assess students’ understanding of conceptual ideas, theoretical concepts, laboratory procedures, and safety protocols before conducting experimental activities. Following each quiz, results were reviewed with students and discussed to address any misconceptions and provide immediate feedback. In Section 3, group presentations and discussions provided a platform for students to share their experimental findings and engage in data analysis, thereby fostering a collaborative learning environment. We have compiled a list of frequently asked questions (FAQs) and corresponding answers that arose during the experiments in the instructor notes (see ). Through the Q&A discussion during experiments, students were able to clarify their understanding of key concepts, such as the role of each component in the synthesis, the impact of oxygen on the products and their properties, and the metal–ligand interactions, while improving their problem-solving and decision-making skills. This facilitated their ability to design and carry out the experiments effectively, think critically about the results, and make informed decisions about their approach.

Upon completion of the experiment, students submitted individual scientific-style reports that included an introduction, methods, results, discussion, and conclusion. These reports assessed students’ comprehension of laboratory procedures, data analysis, and scientific writing skills. To ensure academic integrity, students were required to complete their reports independently, which contributed to their overall grade for the experiment. The report requirements and rubrics are provided in . Instructors provided personalized feedback on writing style and content to each student, discussing areas for improvement, and offering guidance on how to enhance the clarity and coherence of their reports. This laboratory experience is part of the Physical Chemistry Laboratory course, which enrolls 10–12 junior or senior students. Assessment results suggest consistent progress across both reporting tasks with students demonstrating their understanding of core experimental techniques, scientific concepts, and data interpretation skills. Specifically, grades for Part 1 of the reports ranged from 70% to 90%, with an average score of 80%. In Part 2, scores ranged from 75% to 95%, with an average grade of 85%. While limited access to TEM and XRD instrumentation may have impacted students’ understanding of related content, the limitation was mitigated by the emphasis on hands-on data analysis of TEM images and XRD patterns. The overall quality of the reports across both sections suggests that students successfully developed a strong grasp of the airtight synthesis of nonprecious metal nanoparticles, optical characterization of metal nanoparticles, and metal–ligand chelated compounds. This laboratory experience provides a foundational understanding and hands-on training in nanochemistry, preparing students for careers in chemistry and materials science.

## Conclusion

This laboratory experiment provides a unique opportunity for students to learn air-free chemistry techniques while exploring the physicochemical properties of nonprecious metal nanoparticles. The synthesis of copper nanoparticles serves as a benign example, allowing students to master the Schlenk line technique in a safe and controlled environment. Through this experiment, students gain hands-on experience with characterizing nanoparticles using UV–vis spectroscopy and analyzing TEM and XRD data and develop a deeper understanding of their plasmonic properties. Furthermore, the colorimetric method using a Cu-ammonia solution introduces students to crystal field theory and DFT calculation, enabling them to understand the origin of the deep blue color and apply this knowledge to quantitative analysis. This laboratory provides a comprehensive learning experience, integrating theory and practice to give students a solid foundation in nanochemistry and nanomaterial characterization.

## Supplementary Material





## Notes

Laboratory Preparation for Instructors, Instructor Notes, Lab Manuals, and Appendices for TEM and XRD data analysis, DFT calculation, Pre-Lab quizzes, Report Requirement and Rubrics (PDF)
Sample TEM and XRD Data (ZIP)
