# Supplementary material for: Synthesis and Characterization of Copper Nanoparticles: A Laboratory Experiment for Undergraduate Physical Chemistry
Source: J Chem Educ. 2025 Nov 13;102(12):5235–41. doi: 10.1021/acs.jchemed.5c00561 (PMC12874370; doi:10.1021/acs.jchemed.5c00561)
Supplement: Supplementary file 1 [file ed5c00561_si_001.pdf]

Supporting Information

Synthesis and Characterization of Copper Nanoparticles: A Laboratory Experiment for Undergraduate Physical Chemistry

Jonathan Batey, Deborah Okyere, Sarah York, Feng Wang, and Jingyi Chen*

Department of Chemistry and Biochemistry, University of Arkansas, Fayetteville, AR 72701, United States

*Corresponding author: chenj@uark.edu

Table of Contents

1. Laboratory Preparation for Instructors S2
1.1. Chemicals S2
1.2. Materials S2
1.3. Methods S3
1.4. Instrumentation S3
2. Instructor Notes S4
2.1. General Notes S4
2.2. Notes for Section 1 .... S4
2.3. Notes for Section 2 .... S5
2.4. Notes for Section 3 .... S5
2.5. Frequently Asked Questions and Answers S5
3. Laboratory Manual S8
3.1. Laboratory Manual Part I S8
3.2. Laboratory Manual Part II S11
4. Appendix S15
APPENDIX I. TEM and XRD Data Analysis and Sample Results S15
APPENDIX II. DFT Calculation of UV-vis Spectrum S20
APPENDIX III. Sample Pre-Lab Quizzes S22
APPENDIX IV. Report Requirements and Rubrics S24
5. References S26

1. Laboratory Preparation for Instructors

The following information is intended for instructors and teaching assistants (TAs) to prepare for Sections 1 and 2 of the laboratory experiments.

1.1. Chemicals (per group with 2-3 students)

In Section 1, chemicals and materials needed for Cu synthesis and optical characterization: copper 2,4-pentanedionate ($Cu(acac)_2$, 98%) – 55 mg; oleylamine (OLAM, 70%) – 5 mL; ethanol (200 proof) – 30 mL; toluene (ACS) – 10 mL

In Section 2, chemicals needed for Cu quantification: copper (II) chloride ($CuCl_2$, anhydrous, 99%) – 25 mg; ammonium hydroxide (NH_4OH or $NH_3{\cdot}H_2O$, 28 wt.% NH_3) – 2 mL; Ultrapure water with a resistance of 18 MΩ – 50 mL; a NH4OH/water solution at a 1:9 ratio – 30 mL

For our experiment, $Cu(acac)_2$, $CuCl_2$, and $NH_3{\cdot}H_2O$ can be purchased from Thermo Scientific (former Alfa Aesar). OLAM and toluene can be purchased from Millipore-Sigma (former Sigma-Aldrich). Ethanol can be purchased from Koptic through Avantor Sciences (former VWR). All chemicals were used as received. Alternative sources can be used as long as the reagents have the same purity.

1.2. Materials

The following glassware may be required, depending on the specific needs of the experiment: single-manifolds, condensers, reaction flasks, glass stoppers, glass cuvettes, graduated pipettes, and test tubes. All glassware is reusable and should be cleaned and dried after each use to maintain its integrity. Cleaning can be performed as needed, and drying can be done in an oven or by air-drying, depending on the specific requirements of the equipment. Additional necessary equipment includes clamps, vials, syringes, needles, and rubber septa, which should be readily available for use as needed. Inert gases, such as nitrogen or argon, are also required to facilitate the experiment.

1.3. Methods

For Section 1, a single-manifold system should be set up for purging with inert gas. The reaction temperature can be monitored and controlled using one of two configurations: either by inserting a thermocouple into the solution (Figure 2A) or by utilizing a heating mantle without an immersed temperature probe (Figure 5A). Both approaches facilitate easy implementation in various laboratory environments, but must be established beforehand to ensure a successful experiment. The latter setup requires calibration of the solution temperature (Figure 5B) to account for the discrepancy between the set heat mantle temperature and the actual solution temperature.

For Section 2, the necessary equipment and materials should be prepared to facilitate the accurate preparation of working solutions and samples. This includes a graduated pipette or micropipettes (e.g. 1-10 mL, 0.1-1 mL, or 10-200 μL) for accurate transfer of small volumes of liquids, test tubes with caps for mixing and storing working solutions and samples, and plastic cuvettes for holding and analyzing the prepared samples. Additionally, it is recommended to have a pipette tip rack and extra pipette tips on hand to ensure sterility and prevent cross-contamination, as well as a tube rack or test tube holder to organize and secure the test tubes during preparation and analysis.

1.4. Instrumentation

The necessary instrumentation for the experiments includes a balance or scale with sufficient precision (e.g. 0.01g or 0.1mg) for accurately weighing out solid reagents, a centrifuge for efficient separation of solid particles from liquids or suspensions, as well as a UV-Vis spectrophotometer, which should be readily available for conducting the experiments. Prior to use, the spectrophotometer must be turned on and allowed to warm up for a sufficient amount of time (typically 15-30 minutes) to ensure stability and optimal performance.

2. Instructor Notes

General Notes.

To minimize contamination and ensure a smooth experiment, prepare all necessary chemicals in advance, double the required amount, and distribute them among groups in clearly labeled glass vials. Additionally, consider conducting a trial run beforehand to test the experiment's success and have backup samples on hand in case of unexpected issues.

For optimal synthetic results, use clean, dry, and scratch-free glassware to minimize the impact of impurities and nucleation sites on reduction reactions. Clean glassware promptly with concentrated HNO_3 after each use to prevent scratching, and thoroughly rinse stir bars in acid. Handle HNO_3 with caution, using a fume hood and proper disposal procedures (dilution and neutralization with $NaHCO_3$) to ensure safety.

Notes for Section 1.

The synthesis is performed under an inert atmosphere using an air-tight setup with argon or nitrogen. The reaction temperature is regulated by a digital temperature controller, either through a heating mantle or a thermocouple in a glass sleeve. Moderate stirring is maintained, and the reaction mixture is heated from room temperature to 220 °C over 10-15 min, with precise control of the heating rate not being considered necessary. After the reaction, the mixture is removed from the metal container to facilitate cooling. The purification process is carried out by centrifugation at 6000-8000 rcf, with the time being adjusted as needed for effective separation (10-20 min). Centrifuge tubes are filled to no more than 2/3 capacity to ensure efficient separation. If available, TEM and XRD can be used with TA assistance to prepare samples and collect data for Section 3. For TEM, samples are prepared by drop-casting onto a grid. For XRD, sample preparation varies

by instrument, but may involve packing into a capillary tube or drop-casting onto a glass substrate. If instruments are not available, pre-collected data is provided for analysis practice in Section 3.

Notes for Section 2.

Ultrapure water with a resistance of 18 MΩ is used to minimize ion contamination during elemental analysis. Calibration curves are prepared by students, who perform their own calculations for each dilution and use a minimum of 2 mL per dilution. Precise dilutions are achieved by using a balance to measure the volume of water, assuming a density of 1 g/mL. Cu nanoparticles are accurately aliquoted and dried using nitrogen or vacuum during sample preparation. Gentle heating and sonication are used to ensure complete dissolution. Optical measurements are taken using standard 10 × 10 mm plastic cuvettes, with a measurement range of 300 to 800 nm. The cuvettes are thoroughly cleaned between measurements, and readings are taken from most diluted to most concentrated solutions. The background signal is subtracted from the peak intensity for accurate quantification.

Notes for Section 3.

Student presentations are presented as a team for their working group. They can be assigned to present either Section 1 or Section 2. Instructors should encourage students to ask questions to each other. The second half of the section is a lecture of data analysis and interpretation in which students excise the analysis with the provided data.

Frequently Asked Questions and Answers.

Q1. Do I need to measure exactly 54.2 mg of $Cu(acac)_2$ for the Cu synthesis?

A1. No, weighing between 50-60 mg should be fine, but write down the exact weight in the notebook for reference and calculations.

Q2. What rate of inert gas flow should I use to remove air in the reaction flask?

A2. You can judge by the gas bubbler; it's about 1-5 seconds per bubble.

Q3. What should the stirring speed be set at during the synthesis?

A3. The stirring speed should be adjusted to ensure thorough mixing of the solution without splashing. A good starting point is a speed of medium-low setting, but this can be increased as long as splashing is avoided.

Q4. How long does it take to heat the reaction solution to the desired temperature?

A4. It usually takes about 10-15 min depending on the type of heating mantle used.

Q5. Does it matter if we let the reaction proceed for a shorter or longer reaction time?

A5. Yes, it does. This can affect the reaction yield and size distribution. In general, longer reaction time can improve reaction yield and uniform size. However, multiple factors can also influence the product, such as the amount of precursors used, and reaction temperature.

Q6. Do the centrifuge speed and time affect the amount of product we can collect?

A6. Yes, they do. In general, increasing the centrifuge speed and time can increase the amount of product collected, up to a point. We typically aim for 90% precipitation, as achieving 100% separation is challenging.

Q7. Should I use a glass or plastic cuvette to take the UV-vis measurement?

A7. The choice of cuvette material depends on the solvent used. Glass cuvettes are recommended for organic solvents, as they can withstand corrosive or dissolving effects. Plastic cuvettes, on the other hand, are suitable for aqueous solutions.

Q8. What if the copper nanoparticles don't fully dissolve in ammonium hydroxide (NH_4OH)?

A8. Try sonication to make them disperse in solution and react with NH_4OH for a few minutes. If still undissolved, gently heat to 50°C. If necessary, add small amounts of additional NH_4OH (e.g. 10 μL at a time) until fully dissolved.

Q9. How do I measure the diameter of nanoparticles using ImageJ software, and calculate the average diameter and standard deviation?

A9. The detailed step-by-step procedure is included in Appendix I with sample results provided.

Q10. How do I handle particle aggregates on the TEM images?

A10. Aggregates may be either inherent to the product or an artifact of TEM sample preparation. To address this, we recommend manually measuring the size of individual particles that can be clearly identified using ImageJ, while ignoring any ambiguous or aggregated features. This approach allows students to focus on analyzing the size of distinct nanoparticles, minimizing the impact of potential artifacts or aggregates.

Q11. How do I plot the XRD data from the raw files?

A11: To plot the XRD data, obtain the file from the TA or example provided and open it in Excel. Identify the columns for diffraction angle (2θ, degree) and intensity (counts), then create a plot of angle vs. intensity, labeling the axes correctly.

Q12. How do I index the XRD pattern of a substance?

A12. To index an XRD pattern, measure Bragg angles (2θ) and convert to interplanar spacings (d) using Bragg's law. Analyze d-spacing ratios to identify lattice types, then match observed d-spacings with calculated values to assign Miller indices. Successful indexing requires all major peaks to match allowed reflections in a single lattice. For known materials, such as Cu or Cu_2O, in this experiment, compare your data to standard patterns in databases to index the peaks.

3. Laboratory Manual

Copper (Cu) nanoparticles offer a cost-effective and tunable platform for diverse applications, including ultrasensitive sensing, enhanced photocatalysis, and photothermal therapy.[1-3] Despite oxidation challenges, protective strategies can unlock their full potential, positioning Cu as a sustainable alternative to noble metals in diagnostics, energy conversion, and biomedicine. This experiment aims to introduce synthesizing Cu nanoparticles using simplified air-tight methods, evaluating their formation by their optical properties, known as localized surface plasmon resonance (LSPR), and quantifying Cu concentration *via* colorimetry based on the formation of Cu-ammonia complexes. The experiment includes two sections: Part 1. Synthesis and Optical Characterization of Cu Nanoparticles, and Part 2. Quantification of Cu Concentration *via* a Colorimetric Method.

3.1. Part 1. Synthesis and Optical Characterization of Cu Nanoparticles

Introduction

The synthesis of Cu nanoparticles typically involves the reduction of Cu precursors, regardless of the method employed. One of the most widely-used methods is chemical reduction which can occur in different solvent systems, such as water, polyol, and various organic solvents. Notably, oil-based syntheses by oleylamine (OLAM) using air-free chemistry have been found to produce Cu nanoparticles with more uniform size distributions and reduced surface oxidation, making them an attractive option for applications where high-quality nanoparticles are required. OLAM is a versatile reagent commonly employed in oil-based syntheses of inorganic nanoparticles, where it can fulfill multiple roles as a reducing agent, stabilizer, and solvent.[4] In this study, metal nanoparticles of Cu are synthesized through the reduction of copper acetylacetonate, Cu(acac)$_2$, in the presence of OLAM at elevated temperatures. The reaction scheme is illustrated in **Figure S1**. OLAM plays a dual role in this reaction, serving both as a reducing agent and a stabilizer. The reduction process involves a stepwise conversion of Cu(II) to Cu(I) and ultimately to Cu(0), mediated by the formation of intermediate complexes,

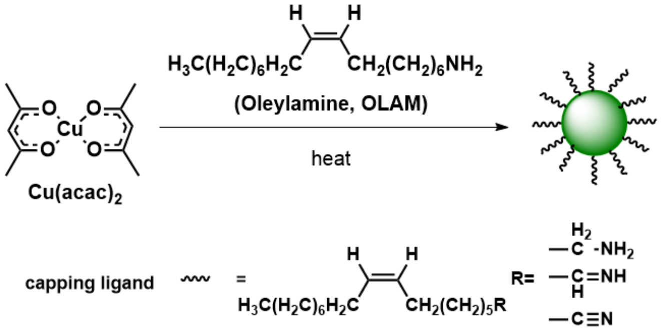

Figure S1. Schematic illustration of the formation of Cu nanoparticles by the OLAM-mediated reduction of Cu precursor.

including Cu(II)-OLAM and Cu(I)-OLAM.[5] The electron transfer from OLAM to Cu(II) and Cu(I) is thought to proceed through the involvement of amino radicals as intermediates, analogous to the reduction of Ag(I) to Ag(0) by OLAM.[6]

The LSPR peak is a collective oscillation of electrons at the surface of metal nanoparticles (**Figure S2A**), which is sensitive to the particle size, shape, and composition.[2] In this case, the sharp and strong LSPR peak indicates that the students have successfully synthesized metallic Cu nanoparticles with limited surface oxidation. Oxidation can lead to a damping of the LSPR peak due to the formation of an oxide layer on the surface of the nanoparticles, which can scatter or absorb the incident light, resulting in a broadened or weakened LSPR peak. The presence of a sharp and strong LSPR peak at 590 nm suggests that the Cu nanoparticles have a relatively clean surface with minimal oxidation (**Figure S2B**), allowing for a strong collective oscillation of electrons and an intense absorption of light at this wavelength.

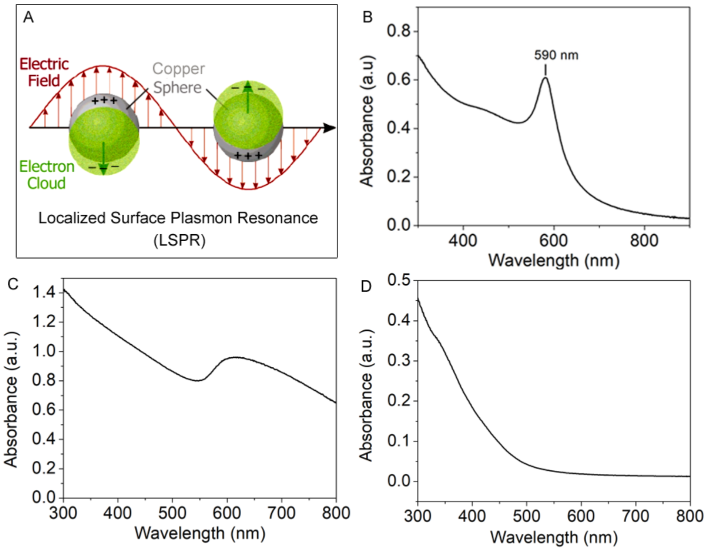

Figure S2. (A) Schematic illustration of LSPR. (B-D) UV-vis spectra of nanoparticles of different composition: (B) Cu, (C) partially oxidized Cu, (D) completely oxidized to Cu_2O.

Oxidation can significantly dampen the plasmonic properties of Cu nanoparticles, leading to a broadening and weakening of the LSPR peak (**Figure S2C**), which can ultimately result in a complete loss of the LSPR feature (**Figure S2D**). This degradation occurs because oxidation forms a layer of copper oxide on the surface of the nanoparticles, which alters their dielectric properties and reduces their ability to support plasmonic excitations. As the oxidation progresses, the LSPR peak shifts to longer wavelengths, becomes less intense, and eventually disappears, indicating a loss of the nanoparticle's plasmonic activity.

Experimental Methods

Safety Notes. When conducting the copper nanoparticle synthesis procedure, prioritize personal safety by wearing lab coats, gloves, and safety goggles. Handle volatile substances such as toluene and ethanol in well-ventilated areas or under fume hoods, and avoid prolonged

inhalation. Exercise caution during high-temperature operations, ensure proper centrifuge tube sealing and balancing, and minimize exposure to toxic substances like toluene.

Synthesis Procedure. The reaction setup is illustrated in **Figure S3**. In a 25 mL three-neck flask equipped with a magnetic stir bar, combine $Cu(acac)_2$ (52.4 mg, 0.2 mmol) and OLAM (5 mL). Connect the flask to a water-cooled condenser and degas the reaction mixture with argon gas for 10 minutes to remove oxygen and moisture. Subsequently, heat the mixture to 220 °C under an inert atmosphere and maintain this temperature for 20 minutes to facilitate the reaction.

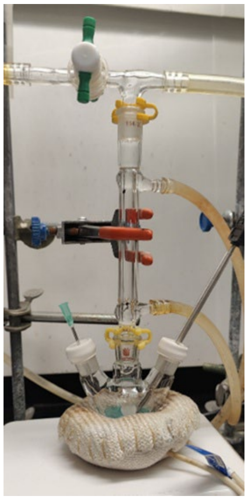

Figure S3. Setup.

Purification Procedure. After completion of the reaction, allow the mixture to cool to 50 °C. Transfer the resulting mixture to a 15 mL centrifuge tube and add a mixture of toluene and ethanol (10 mL, 1:9 v/v). Centrifuge the mixture at 7,500 RCF for 5 minutes to precipitate the product. Collect the product and further purify it through two additional cycles of washing with the toluene-ethanol mixture. Finally, disperse the purified product in 3 mL of toluene for subsequent use.

Optical Characterization. Take a small aliquot (50-200 μL) of the nanoparticle suspension and dilute it to a total volume of 2 mL with solvent in a 1-dram vial. Use toluene as a reference blank to account for any background absorbance. Measure the UV-vis spectrum of the diluted particle suspension over the range of 300-800 nm using a UV-vis spectrophotometer. Record the absorption spectrum, including the wavelength and corresponding absorbance values, and save the data for further analysis. Ensure that the spectrum is properly labeled with the sample identity, concentration, and any other relevant experimental parameters.

Data Process and Analysis

Analyze the UV-vis spectrum of the diluted particle suspension by plotting the wavelength (in nm) on the x-axis and the absorbance on the y-axis. Examine the resulting spectrum closely, paying particular attention to the peak position and shape, which indicate the size distribution and oxidation state of the nanoparticles. Identify a strong, narrow peak in the visible region (around 550-650 nm) as evidence of successful synthesis of Cu nanoparticles with a uniform size distribution. Conversely, recognize a broadened or shifted peak as a potential indication of oxidation of the Cu nanoparticles.

Discussion

Refer to Appendix I and analyze the provided TEM and XRD data in conjunction with your UV-vis spectrum. Compare these data sets to identify correlations or discrepancies between the different characterization techniques. For example, do the TEM images reveal a uniform size distribution consistent with the UV-vis spectrum? Do the XRD patterns indicate the presence of copper oxides, which may be responsible for any shifts or broadening observed in the UV-vis spectrum? What are the implications of these results for the synthesis and characterization of Cu nanoparticles, and how can they be optimized in future experiments? By integrating these data sets, gain a comprehensive understanding of the optical properties and structural characteristics of the Cu nanoparticles.

3.2. Part 2. Quantification of Cu Nanoparticles using a Colorimetric Method

Introduction

Crystal field theory (CFT) is a fundamental concept in chemistry that describes the electronic structure of transition metal complexes. According to CFT, when a transition metal ion is surrounded by ligands, the electrostatic field generated by the ligands splits the metal's d orbitals into different energy levels. The magnitude of crystal field splitting energy (Δ) depends on the type of ligand, its charge, and the metal-ligand distance. By analyzing the crystal field splitting, chemists can predict the electronic configuration, magnetic properties, and spectroscopic behavior of transition metal complexes, providing valuable insights into their chemical reactivity and physical properties.

The energy diagram of octahedral and tetrahedral complexes is presented in **Figure S4**. In an octahedral complex, the six ligands are arranged around the central metal ion in an octahedral geometry. This arrangement leads to a splitting of the d orbitals into two sets: the t_{2g} (d_{xy}, d_{yz}, d_{zx}) and e_g ($d_{x^2-y^2}$, d_{z^2}) sets. The energy difference between these two sets is known as the crystal field splitting energy (Δ_o). In a tetrahedral complex, the four ligands are arranged around the central metal ion in a tetrahedral geometry. This arrangement leads

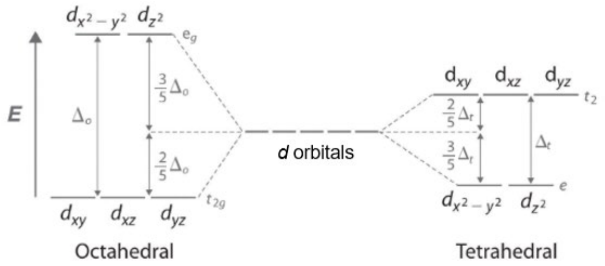

Figure S4. Energy diagram of crystal field splitting without ligands for octahedral and tetrahedral complex.

to a splitting of the d orbitals into two sets: the *e* ($d_{x^2-y^2}$, d_{z^2}) and t_2 (d_{xy}, d_{yz}, d_{zx}) sets. The energy difference between these two sets is also known as the crystal field splitting energy (Δ_t). Δ_t is smaller than Δ_o ($\Delta_t = 0.44\ \Delta_o$) for the same ligands, and is typically smaller than the spin pairing energy. Therefore, tetrahedral complexes are usually high-spin.

The strength of the ligand field determines whether a transition metal complex is classified as a strong field or weak field system. A strong field ligand, such as ammonia (NH_3), carbon monoxide (CO), or cyanide (CN^-), causes a large splitting of the d orbitals, resulting in a significant energy difference between the t_{2g} and e_g sets. In contrast, a weak field ligand, like water (H_2O) or chloride (Cl^-), produces a smaller splitting, with less energy difference between the two sets. Distorting an octahedral complex by moving opposite ligands away from the metal could produce square planar arrangement (**Figure S5**), in which interactions with equatorial ligands become stronger. For example, in the complex $[Cu(NH_3)_4]^{2+}$, the ammonia ligands create a strong field environment, which causes the *d* orbitals to split significantly. As a result, the Cu(II) ion adopts a low-spin configuration, where the electrons occupy the lower-energy t_{2g} orbitals, resulting in a d^9 electronic configuration with one unpaired electron. This configuration favors a square planar geometry, where the four ammonia ligands are arranged around the Cu(II) ion in a plane, with the d_{z^2} orbital pointing along the z-axis and the $d_{x^2-y^2}$ orbital lying in the xy-plane. The resulting geometry is a slightly distorted square plane, with Cu-N bond lengths of approximately 2.06 Å.[9]

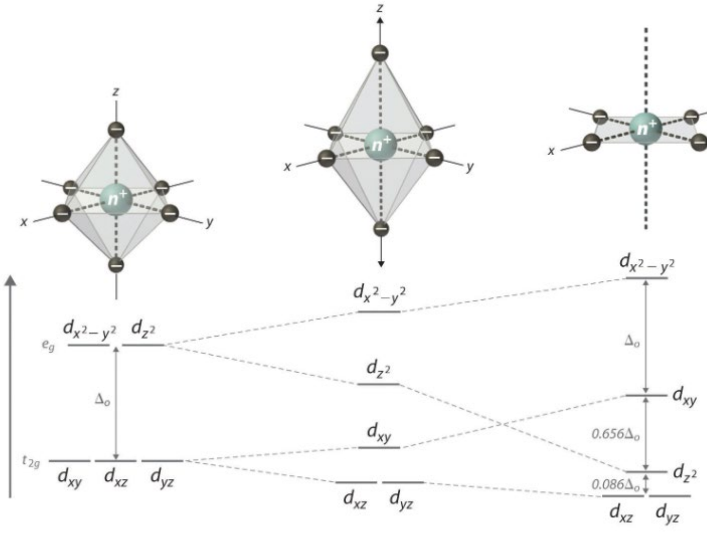

Figure S5. Energy diagram of crystal field splitting from an octahedral, to an elongated octahedral, and a square planar complex.

The crystal field splitting energy is also responsible for the $[Cu(NH_3)_4]^{2+}$ complex's characteristic color. The resulting d-orbital splitting gives rise to characteristic *d-d* transitions in the visible region, which are responsible for the complex's blue color and can be used to probe its electronic structure and bonding properties. When white light is incident on the complex, the

electrons absorb this specific wavelength of light and undergo a *d-d* transition, resulting in the complex appearing blue, as it reflects the complementary color of the absorbed light.[10]

Furthermore, it has been documented that Cu can dissolve in ammonia solutions.[11-12] The dissolution process involves oxygen as follows: Cu(s) + $1/2O_2$(g) + $4NH_3 \cdot H_2O$(l) → $[Cu(NH_3)_4]^{2+}$(aq) + $2OH^-$(aq) + $2H_2O$(l). The kinetics of Cu dissolution for the bulk are largely dictated by the transport of oxygen. In contrast, this limitation is significantly mitigated for Cu nanoparticles due to their high surface-to-volume ratio, which facilitates enhanced oxygen accessibility. Leveraging this property, the dissolution of Cu nanoparticles in ammonia can be effectively combined with the colorimetric method based on the Cu-ammonia complex to quantify the concentration of Cu in these nanoparticles.

Experimental Methods

Safety Notes. When handling ammonia water, exercise caution and follow proper safety protocols to minimize exposure. Wear protective gear, including gloves, goggles, and a face mask, as ammonia can cause skin irritation, eye damage, and respiratory problems. Work in a well-ventilated area, away from heat sources and open flames, as ammonia is highly flammable and can ignite easily. In case of exposure, immediately flush the affected area with water and seek medical attention if necessary. Always handle ammonia water in a fume hood or ventilated enclosure, and dispose of it according to institutional procedures to ensure a safe working environment.

Establishment of Calibration Curve for $[Cu(NH_3)_4]^{2+}$ complex. Establish a calibration curve for the chromophores and determine their molar absorptivity prior to analyzing unknown samples. Prepare a 10 mL Cu stock solution by dissolving 25 mg of $CuCl_2$ in a mixture of $NH_3 \cdot H_2O$ and H_2O, allowing the solution to react to facilitate the formation of the $[Cu(NH_3)_4]^{2+}$ complex. Dilute the stock solution with water to obtain standard solutions of varying concentrations. Measure the absorbance of each standard solution using a UV-Vis spectrophotometer and plot the absorbance values against concentration. Perform linear regression analysis on the data to generate the calibration curve and calculate the molar absorptivity of the $[Cu(NH_3)_4]^{2+}$ complex from the slope of the resulting curve. Use this calibration curve to analyze unknown samples and determine their concentrations.

Dissolution of Cu Nanoparticles. Dry a 200 μL aliquot of Cu nanoparticles suspended in toluene under a gentle nitrogen stream. Add 200 μL of $NH_3 \cdot H_2O$ to the dried Cu nanoparticles to facilitate their digestion. Once digestion is complete, dilute the resulting solution with water to a

total volume of 2 mL. Allow the solution to react for at least 15 minutes to ensure formation of the $[Cu(NH_3)_4]^{2+}$ complex. Measure the absorbance of the solution using a UV-vis spectrophotometer. Calculate the Cu concentration based on the difference in absorbance between 600 nm and 450 nm, utilizing the previously determined molar absorptivity of the $[Cu(NH_3)_4]^{2+}$ complex according to Beer's Law. Use this calculation to determine the Cu concentration in the solution.

Data Process and Analysis

Generate a calibration curve for the colorimetric method by plotting the absorbance of the Cu-ammonia complex at the wavelength of 600 nm as a function of concentration. Determine the molar absorptivity (ε) of the Cu-ammonia complex from the slope of the calibration curve. Then, use the molar absorptivity to calculate the copper concentration in the unknown sample using the Beer-Lambert law. Calculate the reaction yield of your Cu synthesis.

Discussion

Explain the origin of the color of the Cu-ammonia complex. Identify any differences in the UV-vis spectrum of the samples versus those of the standard solutions, and provide an explanation and propose solutions to mitigate the difference or compensate for the difference. From the Cu concentration, estimate the particle concentration. What potential limitations or sources of error may have influenced the results, and how can they be mitigated in subsequent studies?

Theoretical Calculations

If resources are available, a demonstration of DFT calculations of the UV-vis spectrum of the Cu-ammonia complex using Gaussian software can be performed, and the results can be compared to those obtained experimentally.

Appendix I. Data Analysis and Sample Results

This document can serve as a basis for a lecture that incorporates student exercises and discussions on data analysis, provided that time allows for these additional activities.

Introduction

Nanoparticle characterization is commonly performed using transmission electron microscopy (TEM) to examine morphology and x-ray diffraction (XRD) to verify identity based on crystal structure. By combining TEM and XRD techniques, a comprehensive understanding of the nanoparticles' structural properties can be achieved, enabling a thorough characterization of their morphology, composition, and crystal structure.

TEM utilizes a beam of electrons to produce high-resolution images of the internal structure of materials at the nanoscale. By leveraging the wave-particle duality of electrons, TEM enables detailed analysis of nanoparticle morphology, structure, and composition, providing valuable insights into their physical and chemical properties. In this study, we employ the bright-field imaging mode of TEM to capture two-dimensional images of the nanoparticles, which provides information on their size, shape, and distribution. The bright-field mode is particularly useful for visualizing the morphology of nanoparticles, as it produces high-contrast images that highlight the differences in electron density between the particles and the surrounding environment. By analyzing these images, we can gain a deeper understanding of the nanoparticle's dimensions, such as their diameter, length, and aspect ratio, as well as their shape, including any deviations from spherical or other idealized geometries.

The principle of XRD is based on the diffraction of x-rays by the periodic arrangement of atoms in a crystal lattice, described by Bragg's law: $n\lambda = 2d \sin(\theta)$, where n is an integer, λ is the wavelength, d is the interplanar distance, and θ is the angle of incidence. By analyzing the XRD pattern, which consists of peaks corresponding to specific lattice planes, we can determine the crystal structure and lattice parameters of the material. To interpret the pattern, we index the peaks by assigning Miller indices (hkl) based on their position and intensity, considering crystal symmetry and lattice parameters. This process involves identifying major peaks, calculating d-spacings, and verifying the indexing to ensure consistency with known crystal structures. Through XRD analysis, we can gain valuable insights into the crystallinity, structure, and phase purity of nanoparticles, essential for understanding their physical and chemical properties and optimizing their performance in various applications.

Sample Results of UV-vis Data in conjunction with XRD and TEM Data

Samples 1-3 were synthesized under varying conditions with increasing levels of product oxidation. The results, presented in **Figure A1**, combine UV-vis spectroscopy with XRD and TEM data, revealing that changes in the optical properties of the nanoparticles are primarily driven by alterations in their composition. As oxidation increases, the LSPR peak undergoes a redshift, decreases in intensity, and ultimately vanishes, signifying a loss of plasmonic activity in the nanoparticles. This correlation suggests that UV-vis spectroscopy can be employed as a convenient and non-destructive method to monitor the oxidation state and, by extension, the success of the Cu nanoparticle synthesis.

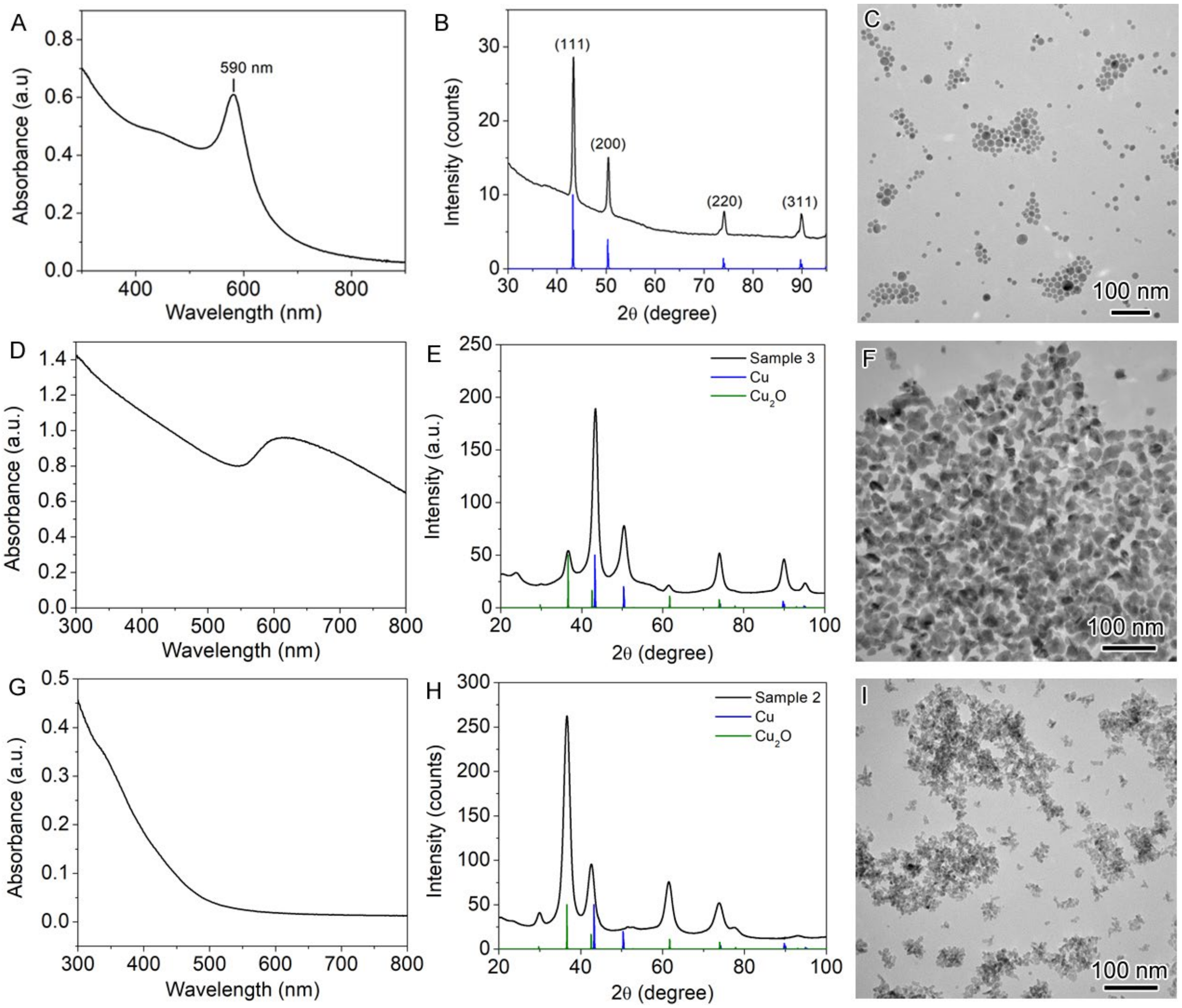

Figure A1. UV-vis, XRD, and TEM characterization of Sample 1, Sample 2, and 3 obtained from air-tight setups synthesized at different reaction conditions: (A-C) 220 °C with argon purging before reaction and protection during reaction, (D-F) 220 °C, with argon purging only, (G-I) 180 °C, without argon purging.

TEM Data Analysis

TEM image analysis is performed using ImageJ software to extract size information from the nanoparticle images. The step-by-step measurement procedure is outlined below, along with sample results to illustrate the process.

1. Open the image to be analyzed in ImageJ.
2. Select "Analyze" and then Set Scale. Under Set Scale, enter the distance in pixels that is measured from the scale bar on the image and enter the known distance to be shown on the TEM image (e.g. 100 nm in **Figure A2**). Change the unit length to nm and check the box for global, and click on OK to have everything set.
3. Use the straight line to measure the diameter of the particles. Use the command control +M to save individual measurements. Under the Results tab, select Summarize to obtain a summary of the data collected.
4. Save the summary data as .csv for opening the data in Microsoft Excel (**Figure A3**).

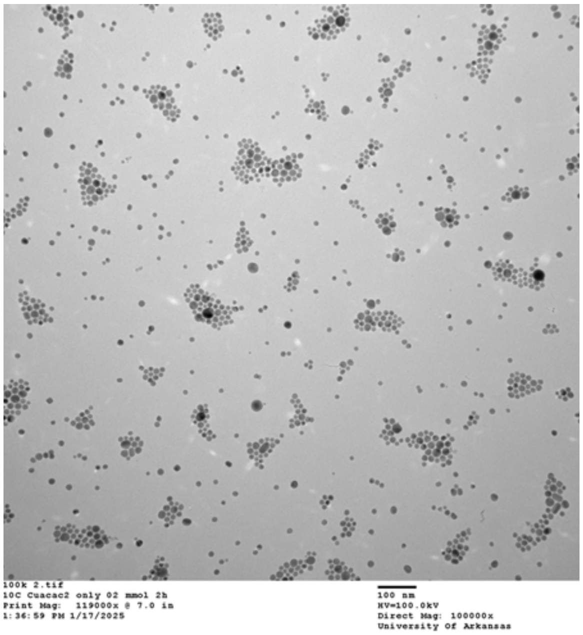

Figure A2. Representative TEM image of nanoparticles, with a scale bar indicating the magnification.

5. Use the measured data to create a histogram of the size distribution of the nanoparticles (**Figure A4**).

	A	B	C	D	E	F	G	H
1		Label	Area	Mean	Min	Max	Angle	Length
2	1		13.889	81.339	68.983	97.083	-38.66	16.008
3	2		15.972	83.414	69.909	104.707	-43.152	18.276
4	3		10.417	103.193	96.796	110.408	-33.69	12.019
5	4		13.889	84.569	56.307	137	-47.121	15.921
6	5		11.806	109.403	100.875	153	-50.194	13.017
7	6		9.028	114.865	109.75	120	-41.634	10.035
8	7		11.806	103.696	85.656	117	-52.431	13.668
9	8		9.722	112.638	106.976	124	-28.61	10.442
10	9		12.5	105.739	100	123	-54.462	14.337
11	10		11.806	91.506	64.137	153	-45	12.964
12	11		18.056	43.177	14.835	99	-58.241	20.582
13	12		15.278	75.737	55	125	-52.595	17.834
14	13		15.278	128.297	113.286	161	-41.186	17.717
15	14		17.361	106.993	96.167	122	-41.634	20.069
16	15		13.194	119.76	87.667	160	-49.399	15.366

41	40		11.806	127.118	119	140	-82.875	13.437
42	41		15.278	118.438	102.878	136	-62.241	17.892
43	42		18.75	100.556	90.852	121	-67.38	21.667
44	43		10.417	110.541	101	129	-126.027	11.335
45	44		11.806	118.76	111.062	143	-42.51	13.566
46	45		14.583	83.126	69.9	121	-63.435	16.771
47	46		13.194	120.031	108.074	171	-51.843	14.837
48	47		10.417	139.581	131	144.071	-30.256	11.577
49	48		8.333	122.235	114.636	133	-74.745	9.501
50	49		8.333	118.937	102.421	157	-45	9.428
51	50		9.028	135.135	124	152	-14.036	10.308
52	51	Mean	13.514	106.772	92.318	134.733	-53.648	15.409
53	52	SD	2.796	20.397	25.049	20.932	22.743	3.351
54	53	Min	8.333	43.177	14.835	97.083	-129.094	9.428
55	54	Max	19.444	139.963	131	188	-14.036	22.189

Figure A3. Summary of the size measurement based on Figure 2 using ImageJ.

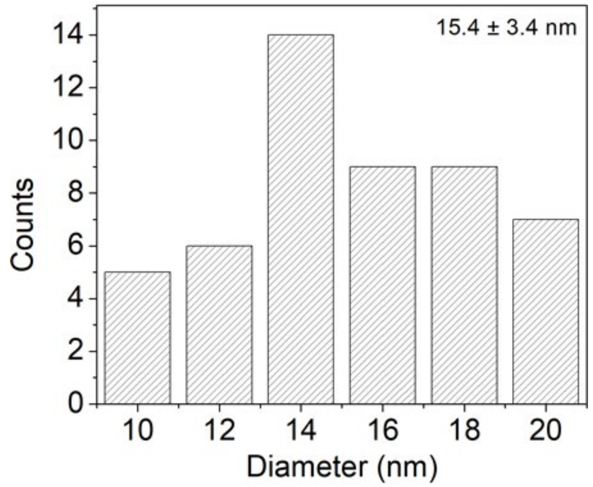

Figure A4. Sample histogram plot of the size distribution based on the data summary in Figure 2.

XRD Data Analysis

Open the XRD data file and import it into a graphing software, such as Excel. Plot the intensity of the diffracted radiation against the diffraction angle (2θ) to generate the XRD pattern. Ensure that the plot is properly labeled with title, axis labels, and units. The resulting plot should display a series of peaks corresponding to the different crystal planes in the sample. Identify the major peaks in the pattern and record their corresponding 2θ values. Use a crystallographic database, such as the American Mineralogist Crystal Structure Database, to search for peak patterns of face-centered cubic (FCC) Cu.[7-8] Compare the observed peak positions with those predicted by the database for different crystalline phases. Assign Miller indices (*hkl*) to each peak based on the best match, considering the symmetry and lattice parameters of the crystal structure. An example is shown in **Figure A5**.

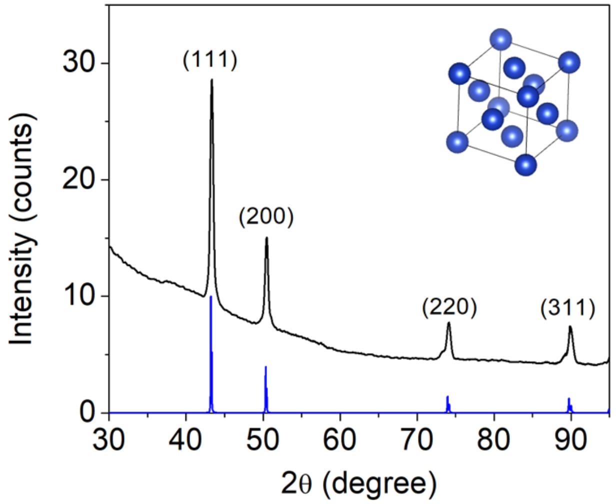

Figure A5. The observed XRD pattern (black) is indexed to the corresponding to that of Cu fcc structure (blue). The inset is the fcc unit cell with a lattice constant of 3.615 Å. The assigned indices satisfy the Bragg equation: $n\lambda = 2d(hkl)\sin(\theta)$, where n is an integer, λ is the wavelength of the incident radiation, *d(hkl)* is the interplanar spacing, and θ is the diffraction angle.

Calculation of Particle Concentration

Estimate the concentration of metal nanoparticles using results from Section 1 and 2. The calculation method, applicable to metallic nanoparticles with FCC structures, provides a general overview of particle concentration estimation. The particle concentration ($C_{particle}$, particles/mL) can be calculated using equation (3):

$$C_{particle} = C_{Cu} / M_{CuNP} \qquad (3)$$

where C_{Cu} is the concentration of elemental Cu and M_{CuNP} is the mass per Cu nanoparticle. M_{CuNP} can be calculated based on TEM image analysis, as detailed in Appendix I. Equations (4-6) provide additional steps for calculating particle volume, mass, and molar concentration. The volume of a spherical particle (V, nm) with a radius of r (nm) can be obtained by equation (4):

$$V = \tfrac{4}{3}\pi r^3 \qquad (4)$$

The mass per Cu nanoparticle (M_{CuNP}, g) can then be calculated using equation (5):

$$M_{CuNP} = V / a^3 \times N \times M_{Cu} \qquad (5)$$

where a is the lattice constant of Cu (0.361 nm),[20-21] N is the number of atoms per FCC unit cell (4 atoms), and M_{Cu} is the atomic mass of Cu (63.5 amu or 1.055×10^{-22} g per atom) . The particle concentration can be converted to molar concentration ($C_{M,particle}$) using equation (6):

$$C_{M,particle} = C_{particle} / N_a \qquad (6)$$

Appendix II. DFT Calculation of UV-vis Spectrum of Cu-Ammonia Complex

This document can serve as a resource for a lecture that incorporates group exercises and discussions, including a demonstration of DFT calculations of UV-vis spectra using Gaussian 16 software, provided that the necessary computational resources are available.

Background

Density Functional Theory (DFT) is a computational method used to study the behavior of many-electron systems. Rather than solving the many-electron Schrödinger's equation, DFT solves the Kohn-Sham equation, where the exact electron exchange and correlation effects are approximated by an exchange-correlation functional that depends only on ground-state electron density.

The Becke's 3-parameter Lee-Yang-Parr (B3LYP) hybrid functional employed in this study offers an improved prediction of exchange effects by combining the exact exchange from Hartree-Fock theory with density-based DFT exchange, thereby yielding a more accurate estimation of true exchange-correlation energy.

To compute UV-vis spectra, one of the most accurate and efficient approach is time-dependent DFT, where the response of the ground state density to a time-depend perturbation similar to an applied electric field is computed. The poles in such a response give the excitation energies in the absorption spectrum.

Gaussian 16 is a popular computational chemistry software package that enables researchers to perform DFT calculations, such as energy minimizations and determination of UV-vis spectra. The software supports a wide range of exchange-correlation functionals, basis sets, and solvent models, enabling users to tailor their calculations to specific systems and properties of interest.

Simulation Procedure

To determine the UV-vis spectrum, the $[Cu(NH_3)_4]^{2+}$ complex is first optimized in the gas phase using the Gaussian 16 package. First, the molecular structure of the complex is built using the GaussView graphical user interface. By default, the four NH_3 molecules could have a high degree of symmetry with hydrogens opposing each other. It is challenging to perform geometry optimization to find the true minimum when the initial geometry is of high symmetry. Therefore, it is recommended random rotations of the NH_3 molecules to be applied to the initial conformation, which can be easily accomplished using the "Modify Dihedral" tool of GaussView.

The initial conformation is optimized using B3LYP[13] with the aug-cc-pVDZ basis set.[14-15] The optimized conformation is then opened in GaussView to perform a TD-DFT calculation with "NStates=10", which requires 10 excited states to be calculated. In addition, the UV-vis calculation is performed with "scrf=(solvent=water)", which requests the solvents be approximated using the self-consistent reaction field (SCRF) method. The dielectric constant of the environment in the SCRF will be chosen to be that of liquid water.

When visualizing the UV-vis spectra in GaussView, it is important to adjust the range of the y-axis, as the peak at 610 nm is very weak compared to the larger peaks at wavelengths less than 200 nm. The stronger peaks are not measured in experiments as the cuvette used have absorption cutoff below 300 nm. The UV-vis spectrum was exported from GaussView and plotted with the Origin software as shown in Figure 9 of the manuscript.

Appendix III. Sample Pre-Lab Quizzes

Pre-lab quizzes are designed to assess students' understanding of theoretical concepts, laboratory procedures, and safety protocols before conducting an experiment or lab activity. They ensure student preparedness, reinforce theoretical concepts, promote active learning, enhance laboratory safety, and streamline lab activities. Brief 15-min quizzes at the beginning of Sections 1 and 2 assessed students' preparation for lab experiments. Following each quiz, results are reviewed with students and discussed to address any misconceptions and provide immediate feedback. Below is the list of sample pre-lab quizzes.

Conceptual Understanding Question:

Q0. How do the unique properties of metal nanoparticles enable their use in various technological and industrial applications, and what factors influence their performance in these contexts?

Part I. Synthesis and Characterization of Cu Nanoparticles

Q1. What is the primary method used to synthesize Cu nanoparticles in this experiment, and what role does oleylamine (OLAM) play in the reaction?

Q2. Describe the reduction process involved in the synthesis of Cu nanoparticles using OLAM.

Q3. How do you expect the localized surface plasmon resonance (LSPR) peak to appear in the UV-vis spectrum if the Cu nanoparticles are successfully synthesized with minimal oxidation, and what wavelength range would you expect to see it in?

Q4. What is the purpose of degassing the reaction mixture with argon gas before heating it to 220°C, and how does this step contribute to the synthesis of high-quality Cu nanoparticles?

Q5. How do you plan to purify the resulting Cu nanoparticle suspension using a combination of centrifugation and washing with a toluene-ethanol mixture?

Part 2. Quantification of Cu Nanoparticles using a Colorimetric Method

Q1. What is the principle behind the colorimetric method used to quantify Cu nanoparticles, and how does it relate to the formation of the Cu-ammonia complex?

Q2. Describe the dissolution process of Cu nanoparticles in ammonia solutions, including the role of oxygen and the resulting chemical equation.

Q3. How do you plan to establish a calibration curve for the $[Cu(NH_3)_4]^{2+}$ complex using a UV-vis spectrophotometer, and what information will this curve provide for analyzing unknown samples?

Q4. What is the purpose of measuring the absorbance of the solution at 600 nm and 450 nm in the colorimetric method, and how will you use these values to calculate the Cu concentration according to Beer's Law?

Q5. How do you plan to estimate the particle concentration from the Cu concentration obtained using the colorimetric method, and what assumptions will you need to make in this calculation?

Safety and Laboratory Procedures

Q1. What personal protective equipment (PPE) should be worn when handling volatile substances such as toluene and ethanol, and why is it important to work in a well-ventilated area or under a fume hood?

Q2. Describe the proper procedure for handling ammonia water, including any necessary safety precautions and emergency procedures.

Q3. What are the potential hazards associated with working with high temperatures and toxic substances in this experiment, and how can you minimize these risks through careful planning and execution?

APPENDIX IV. Report Requirements and Rubrics

Report Requirements

The objective of writing a lab report is to summarize one's experimental results in compact form, draw conclusions, and communicate results to others. By writing the report, you are using your data to answer specific questions. In the process, you will gain mastery of the relevant theory and also learn some data deduction techniques of general scientific applicability.

The best preparation for scientific writing is to read articles in leading journals, such as the Journal of the American Chemical Society. A good reference is M. Alley, The Craft of Scientific Writing (Prentice-Hall, 1987). The ACS publishes a style guide that is available in the library, and all ACS journals have documents containing "Information for Authors."

The lab manual has explicit instructions as to what needs to be reported, but all reports must contain the following general sections:

1. **Title, with your name and date.**
2. **Abstract:** A brief summary (150-250 words) of what was done, how it was done, and major results. (10/100)
3. **Introduction:** A brief but concise introduction (500-1000 words) to the background and theory. (10/100)
4. **Experimental Method:** A brief description of the method and apparatus, including sketches if applicable. (10/100)
5. **Results:** Data presented in tabular and graphical forms that are easy to work with and clear to the reader. This section should include result description, explanation, and error analysis. Sample calculations should be included in the Appendices. (30/100)
6. **Discussion:** Answers to specific questions being asked in the lab manual, comments on the experiment, sources of error, importance, etc. (30/100)
7. **Appendixes:** a) Carbon copies or photocopies of your data from your lab notebook should be attached to the lab report if applicable; and b) sample calculations. (10/100)

<u>Graphs</u>. All graphs must conform to standard conventions of scientific journals. Points must be large and visible, with a scale chosen so that points are well spread on the page. The independent variable must be on the X-axis. Programs such as Excel provide all the options needed to produce journal-quality graphs.

Lab Report Rubrics

Section	Excellent	Good	Fair	Poor	Points
**Abstract**	Provides a clear, concise, and comprehensive summary, including background, methodology, major results, and conclusions. The abstract is well-written, easy to understand. (9-10 points)	Provides a good summary, but may lack clarity or concision in one or two areas. The abstract is generally well-written. (7-8 points)	Provides a fair summary, but lacks detail or clarity in several areas. The abstract may be poorly written or exceed the word limit significantly. (4-6 points)	Fails to provide an adequate summary, or is missing altogether. (0-3 points)	/10
**Introduction**	Provides a clear, concise, and well-organized introduction to the background and theory. The introduction is well-written, easy to understand, and within the word limit. (9-10 points)	Provides a good introduction to the background and theory, but may lack clarity or concision in one or two areas. The introduction is generally well-written. (7-8 points)	Provides a fair introduction to the background and theory, but lacks detail or clarity in several areas. The introduction may be poorly written or exceed the word limit significantly. (4-6 points)	Fails to provide an adequate introduction to the background and theory, or is missing altogether. (0-3 points)	/10
**Experimental Method**	Provides a clear, concise, and well-organized description of the experimental method. The description is easy to follow. (9-10 points)	Provides a good description of the experimental method, but may lack clarity or concision in one or two areas. (7-8 points)	Provides a fair description of the experimental method, but lacks detail or clarity in several areas. The description may be poorly written or difficult to follow. (4-6 points)	Fails to provide an adequate description of the experimental method, or is missing altogether. (0-3 points)	/10
**Results**	Presents data in a clear, concise, and well-organized manner, using tables, graphs, and figures as necessary. The results section includes a thorough description, explanation, and error analysis of the data. (27-30 points)	Presents data in a good manner, but may lack clarity or concision in one or two areas. The results section may not include all necessary details or analyses. (23-26 points)	Presents data in a fair manner, but lacks detail or clarity in several areas. The results section may be poorly written or difficult to follow. (17-22 points)	Fails to present data in an adequate manner, or is missing altogether. (0-16 points)	/30
**Discussion**	Provides a clear, concise, and well-organized discussion of the results, including answers to specific questions asked in the lab manual. The discussion includes comments on the experiment, sources of error, importance, and relevance to the field. (27-30 points)	Provides a good discussion of the results, but may lack clarity or concision in one or two areas. The discussion may not include all necessary details or analyses. (23-26 points)	Provides a fair discussion of the results, but lacks detail or clarity in several areas. The discussion may be poorly written or difficult to follow. (17-22 points)	Fails to provide an adequate discussion, or is missing altogether. (0-16 points)	/30
**Appendixes**	Includes all necessary appendices, such as carbon copies or photocopies of data from the lab notebook, and sample calculations. The appendices are well-organized and easy to follow. (9-10 points)	Includes most necessary appendices, but may be missing one or two items. The appendices are generally well-organized, but may lack clarity in one or two areas. (7-8 points)	Includes some necessary appendices, but lacks detail or organization in several areas. The appendices may be poorly written or difficult to follow. (4-6 points)	Fails to include necessary appendices, or includes irrelevant information. (0-3 points)	/10
**Total**					/100

References:

1. Li, Z.; Chang, S.; Khuje, S.; Ren, S., Recent Advancement of Emerging Nano Copper-Based Printable Flexible Hybrid Electronics. *ACS Nano* **2021**, *15*, 6211-6232.
2. Kim, S.; Kim, J.-M.; Park, J.-E.; Nam, J.-M., Nonnoble-Metal-Based Plasmonic Nanomaterials: Recent Advances and Future Perspectives. *Advanced Materials* **2018**, *30*, 1704528.
3. Gawande, M. B.; Goswami, A.; Felpin, F.-X.; Asefa, T.; Huang, X.; Silva, R.; Zou, X.; Zboril, R.; Varma, R. S., Cu and Cu-Based Nanoparticles: Synthesis and Applications in Catalysis. *Chemical Reviews* **2016**, *116*, 3722-3811.
4. Mourdikoudis, S.; Liz-Marzán, L. M., Oleylamine in Nanoparticle Synthesis. *Chemistry of Materials* **2013**, *25*, 1465-1476.
5. Jeong, S.; Liu, Y.; Zhong, Y.; Zhan, X.; Li, Y.; Wang, Y.; Cha, P. M.; Chen, J.; Ye, X., Heterometallic Seed-Mediated Growth of Monodisperse Colloidal Copper Nanorods with Widely Tunable Plasmonic Resonances. *Nano Letters* **2020**, *20*, 7263-7271.
6. Chen, M.; Feng, Y.-G.; Wang, X.; Li, T.-C.; Zhang, J.-Y.; Qian, D.-J., Silver Nanoparticles Capped by Oleylamine: Formation, Growth, and Self-Organization. *Langmuir* **2007**, *23*, 5296-5304.
7. Wyckoff, R. W. G., *Crystal Structures 1*, Second Edition ed.; Interscience Publishers: New York, 1963.
8. https://rruff.geo.arizona.edu/AMS/minerals/copper American Mineralogist Crystal Structure Database. (accessed 2/20/2025).
9. Cotton, F. A.; Wilkinson, G.; Murillo, C. A.; Bochmann, M., *Advanced Inorganic Chemistry*; John Wiley & Sons, 1999, p pp865.
10. Copper from Https://www.youtube.com/@chemrulesuark. (accessed 2/19/2025).
11. Halpern, J., Kinetics of the Dissolution of Copper in Aqueous Ammonia. *Journal of The Electrochemical Society* **1953**, *100*, 421.
12. Luo, Q.; Mackay, R. A.; Babu, S. V., Copper Dissolution in Aqueous Ammonia-Containing Media During Chemical Mechanical Polishing. *Chemistry of Materials* **1997**, *9*, 2101-2106.
13. Becke, A. D., Density-Functional Thermochemistry. Iii. The Role of Exact Exchange. *The Journal of Chemical Physics* **1993**, *98*, 5648-5652.
14. Dunning Jr, T. H., Gaussian Basis Sets for Use in Correlated Molecular Calculations. I. The Atoms Boron through Neon and Hydrogen. *The Journal of Chemical Physics* **1989**, *90*, 1007-1023.
15. Kendall, R. A.; Dunning Jr, T. H.; Harrison, R. J., Electron Affinities of the First-Row Atoms Revisited. Systematic Basis Sets and Wave Functions. *The Journal of Chemical Physics* **1992**, *96*, 6796-6806.
