# Supplementary figures and images for: Synthesis and Characterization of Copper Nanoparticles: A Laboratory Experiment for Undergraduate Physical Chemistry
Source: J Chem Educ. 2025 Nov 13;102(12):5235–41. doi: 10.1021/acs.jchemed.5c00561 (PMC12874370; doi:10.1021/acs.jchemed.5c00561)

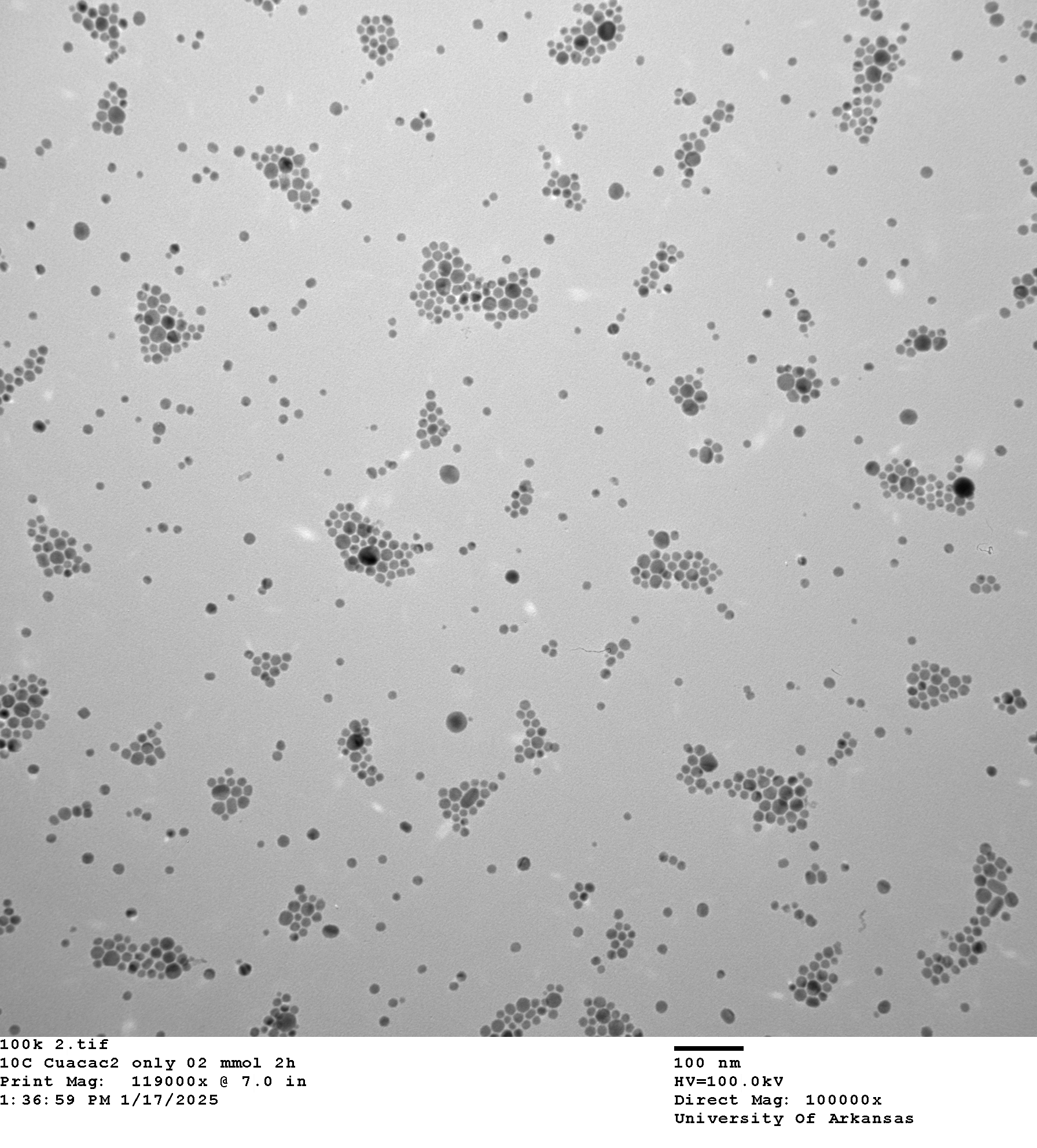

Supplement: Supplementary file 2 [file ed5c00561_si_002.zip › sample data/Sample 1_TEM.tif]

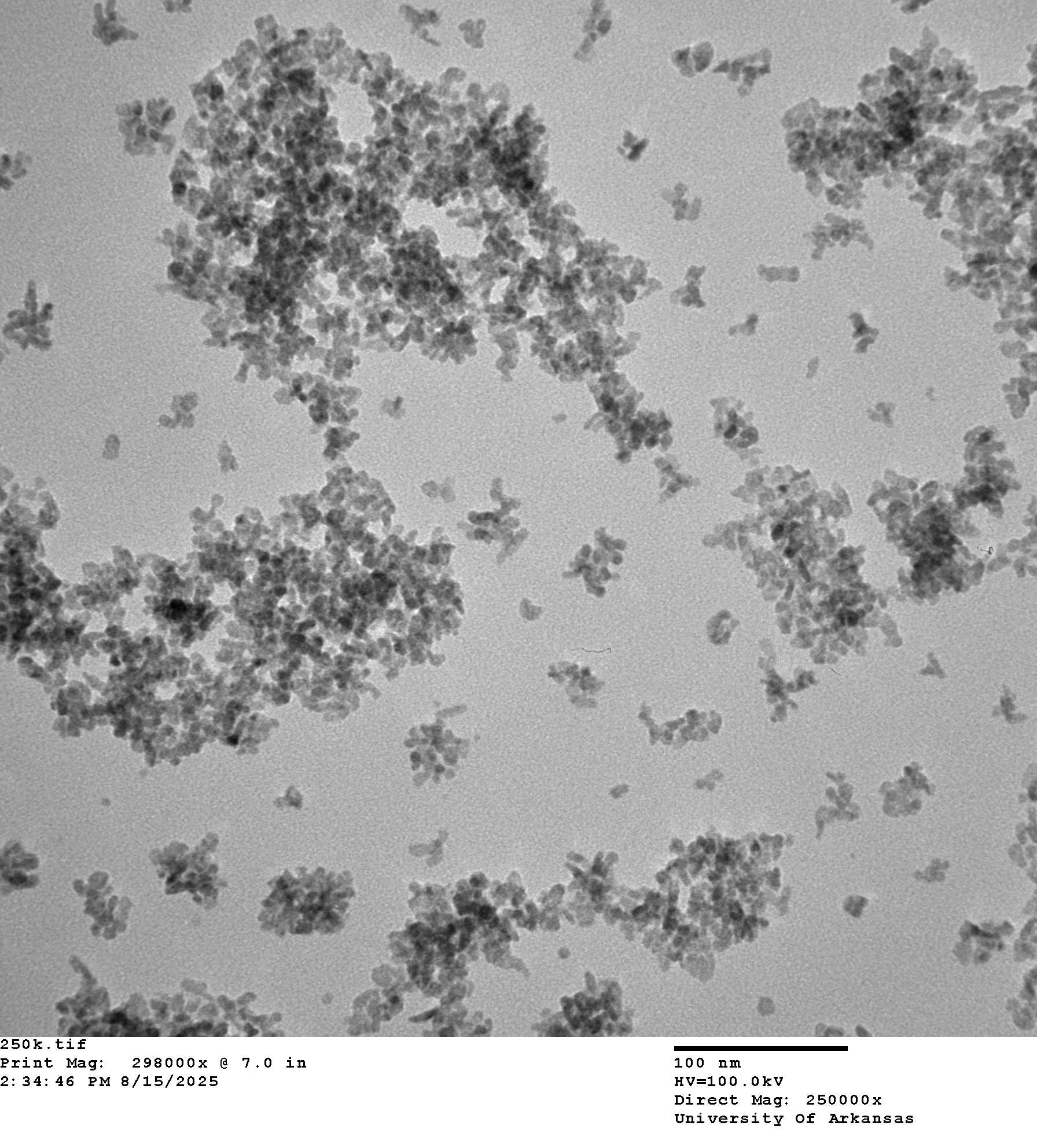

Supplement: Supplementary file 2 [file ed5c00561_si_002.zip › sample data/Sample 2_TEM.tif]

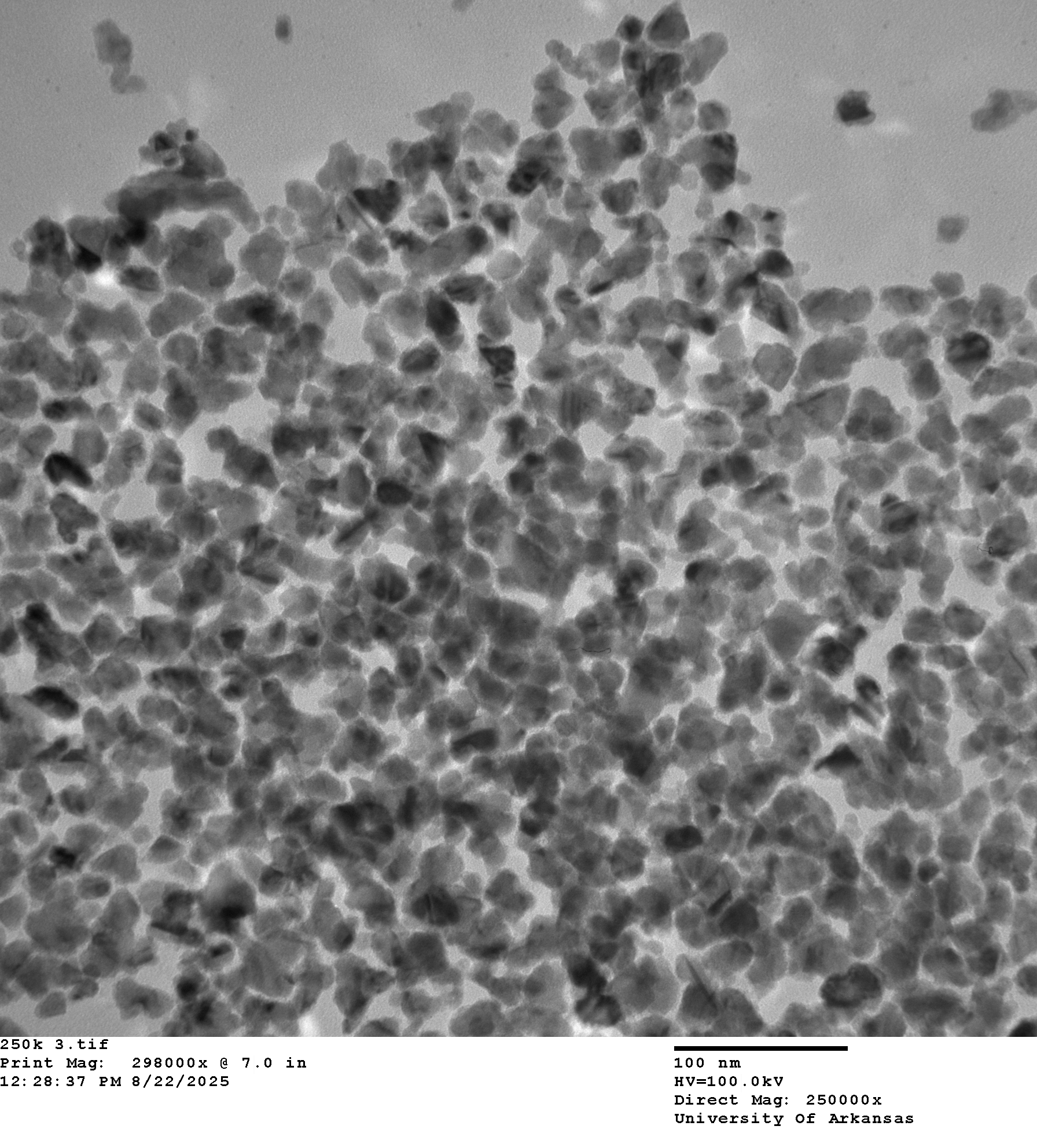

Supplement: Supplementary file 2 [file ed5c00561_si_002.zip › sample data/Sample 3_TEM.tif]
